# Fireproof Nanocomposite Polyurethane Foams: A Review

**DOI:** 10.3390/polym15102314

**Published:** 2023-05-15

**Authors:** Kirill Cherednichenko, Dmitry Kopitsyn, Egor Smirnov, Nikita Nikolaev, Rawil Fakhrullin

**Affiliations:** 1 Department of Physical and Colloid Chemistry, Faculty of Chemical and Environmental Engineering, National University of Oil and Gas “Gubkin University”, Moscow 119991, Russia; kopicin.d@inbox.ru (D.K.); kazanbio@gmail.com (R.F.); 2 Institute of Fundamental Medicine and Biology, Kazan Federal University, Kreml Uramı 18, Kazan 420008, Russia

**Keywords:** polyurethane foams, nanocomposite, nanoparticles, carbon nanomaterials, clay nanomaterials, fire resistance

## Abstract

First introduced in 1954, polyurethane foams rapidly became popular because of light weight, high chemical stability, and outstanding sound and thermal insulation properties. Currently, polyurethane foam is widely applied in industrial and household products. Despite tremendous progress in the development of various formulations of versatile foams, their use is hindered due to high flammability. Fire retardant additives can be introduced into polyurethane foams to enhance their fireproof properties. Nanoscale materials employed as fire-retardant components of polyurethane foams have the potential to overcome this problem. Here, we review the recent (last 5 years) progress that has been made in polyurethane foam modification using nanomaterials to enhance its flame retardance. Different groups of nanomaterials and approaches for incorporating them into foam structures are covered. Special attention is given to the synergetic effects of nanomaterials with other flame-retardant additives.

## 1. Introduction

### 1.1. Polyurethane and Polyurethane Foams

Polyurethane (PU) is a polymer material employed in fabrication of modern, versatile, and safe consumer and industrial environmentally friendly products. Currently, PU is a commercially available strategic material widely used in a number of industrial areas [1] resulting in a continuously increasing PU market [2]. For instance, in 2016, worldwide PU consumption was 9% of the global consumption of plastics [3], and according to recent reports, PU foams (PUFs) are the main component of polymer production [4].

**Figure 1:**
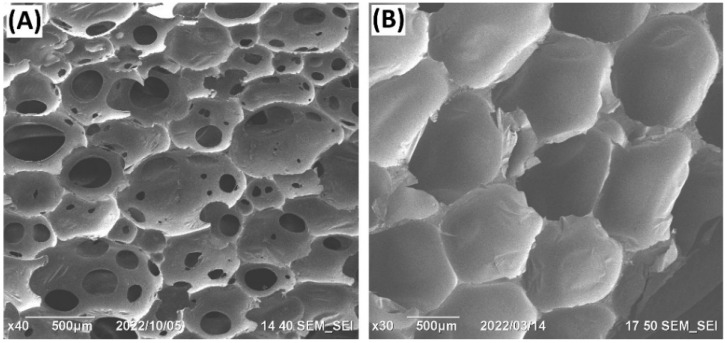
Typical scanning electron microscopy: (**A**) (SEM micrographs of open-cell PUF (FlexFoam-iT! III); (**B**) Sem micrograph of closed-cell PUF (Foam-iT! 3). The images are collected with a JEOL JIB 4501 multibeam system at accelerating voltage of 10 kV and spot size 50.

Foam is a complex material that contains pores/hollows formed by gas in a solid polymer matrix. The microstructure and rheology of foam have significant impacts on the final product properties [5]. PUFs can be classified into flexible (Figure 1A) and rigid (Figure 1B) PUFs, which is the most popular and widespread classification. Nevertheless, alternative classifications such as division according to the cell size can also be applied [6,7,8].

PUF synthesis is a complex and dynamic phenomenon that combines several processes including polymerization and blowing (foam expansion). Polymerization occurs during the exothermic reaction between hydroxyl groups in polyol and isocyanate groups in isocyanate/diisocyanate (reaction of step polymerization) [6,7,9,10]. Methylene diphenyl diisocyanate (MDI), toluene diisocyanate (TDI), and their derivatives are the most widely used isocyanates [11], whereas hydroxyl-terminated polyethers and polyesters are the most frequently used polyols [5,6,7]. The properties of the product obtained dramatically depend on the balance of hard and soft segments of PU chain presented usually by aromatic-based diisocyanate and polyether or polyester polyol, respectively. The “hardness” of hard segments is due to hydrogen bonding between the urethane/urea groups. Thus, for instance, the presence of hard segments makes the foam more rigid, whereas soft segments contribute to its elasticity [12,13,14,15].

Foam expansion is accomplished by introducing blowing gas (a discontinuous phase) into a polymer matrix (a continuous phase), either by adding a chemical blowing agent (e.g., H_2_O, basic acids, enolizable organic compounds) to provide the chemically produced CO_2_ or N_2_ or by adding physical blowing agent/volatile additives with a low boiling point (e.g., chlorofluorohydrocarbons, hydrofluorocarbons, acetone, hexane, pentane, and methylene chloride) [12,16,17,18]. Since polymerization and blowing occur simultaneously, the role of the catalyst (usually presented by amines or tin [12]) to maintain the proper balance between these processes cannot be overestimated [6].

In addition to varying polyol and diisocyanate as well as their starting ratios, there are many ways to significantly control PUF properties. For instance, the polyol molecule size (hence, its flexibility) and number of reactive hydroxyl groups per molecule ultimately control the degree of cross-linking between chains, and hence, the rigidity of the foam. Of note, a higher degree of cross-linking results in higher rigidity. The additional cross-links can be created by cross-linkers and chain extenders [16,19]. Obviously, the synthesis conditions including temperature and humidity as well as synthetic techniques, for example, solution casting, precipitation, and in situ synthesis, significantly influence the resulting PUF properties [6,7,9,10,19,20,21].

As a result of such a wide choice of initial reagents and synthetic approaches to PUF synthesis, a great number of foam materials with various properties have been produced, which, in turn, has made them indispensable in many fields of industry and consumer products. Properties such as low weight, sound and energy insulation, high tensile strength, and easy processability have made PUF a popular material in the production of thermal isolation and building materials [12,16,22,23]. Obviously, due to its porous structure, PUF can be used as an effective sorbent (e.g., at oil spill sites, sewage treatment, and CO_2_ capture [19,24,25]). Moreover, owing to high PU biocompatibility [26], its foams have been used for certain applications in biomedicine [27,28,29]. Viscoelastic PUFs exhibit “shape memory” properties and are widely applied in bedding, furniture, shoe production, as well as aerospace and automotive industries [16,30]. More detailed descriptions of PUF applications in industry have been covered in recent reviews [12,16,19].

However, despite a high prevalence of PUFs in household consumption and industry, their application is still hindered by some particular disadvantages. Arguably, the most serious problem with PUFs is that they are highly flammable, which is accompanied by emission of extremely toxic and combustible gases (e.g., CO and HCN) and smoke [19,31,32,33].

PUF burning is a complex process that includes various stages. However, the three main stages are: thermal decomposition, ignition, and combustion [34,35]. PUF ignition is preceded by thermally induced dissociation of various chemical bonds at different temperatures (first, in hard segments of PU chain, then, in soft segments) and formation of flammable gases and radicals [32,35,36]. Ignition happens after interdiffusion of the former with air [35]. The combustion occurs only in the presence of a sufficient amount of oxygen [34] and is accompanied by the formation of gaseous (combustible and noncombustible gases) and solid (entrained solid particles and carbonaceous char) products [32,33]. These products vary with the foam composition and density, temperature level, rate of temperature rise, and volatile evolution. The flammability of PUFs is stimulated by the increased temperature of the formed gases, and hence, increasing heat transfer from the combustion zone to the adjacent material. This maintains further PU decomposition and ignition which results in flame propagation [32]. High-temperature dissociation of the chemical bonds is accompanied by the formation of free radicals which maintains and enhances the burning process. Obviously, flexible open-cell PUFs are prone to faster fire spread due to the comparatively free transfer of the air inside the foam [35]. Noteworthy, PUF density is another important factor in flame propagation [37].

### 1.2. Fire Retardants

Fortunately, the poor PUF fire resistance can be overcome by the application of fire/flame retardants (FRs) [12,31,32,33,35,38,39,40,41]. FRs are an essential component of commercial, modern PUF-based materials for industrial applications. In particular, there is interest in enhancing PUF fire resistance which is illustrated by the significant number of reviews on this topic [31,32,33,35,38,40,41,42,43,44,45,46].

Historically, halogen-containing compounds were the first FRs applied to PUFs [37]. Nevertheless, the deterioration of PUF mechanical properties and associated environmental issues have led to the emergence of halogen-free FRs based on phosphorus and nitrogen organic and inorganic compounds [31,32,33,35,38,40,41,43,46]. These compounds feature even higher fire retardancy than halogen-containing FRs; however, they are not free from certain drawbacks (e.g., high hygroscopicity of some phosphorous-containing FRs significantly sophisticates fire-resistant PUFs) [35,40,43]. Some of the current FRs that are most widely used are summarized in Table 1.

Various FRs work differently depending on their nature and the method used for their integration into foam [44,45,46,47,48]. FR activities include physical and chemical processes, or their combination. Physical processes include the melt-drop effect, FR’s endothermic break down at high temperatures leading to overall temperature decrease (aluminum/magnesium hydrates [49]), and thermal shielding via formation of a char layer isolating unburned polymer from fire propagation (intumescents, such as expandable graphite [50]). Meanwhile, chemical processes include emission of nonflammable gases (CO_2_ or N_2_) that dilute O_2_ and other flammable gases and slow down burning (melamine [43]) and gas phase radical quenching which implies the generation of FR’s radicals that capture highly reactive H· and OH· radicals in the flame, and hence, retard PUF radical oxidation (DOPO derivatives [51]).

**Figure 2:**
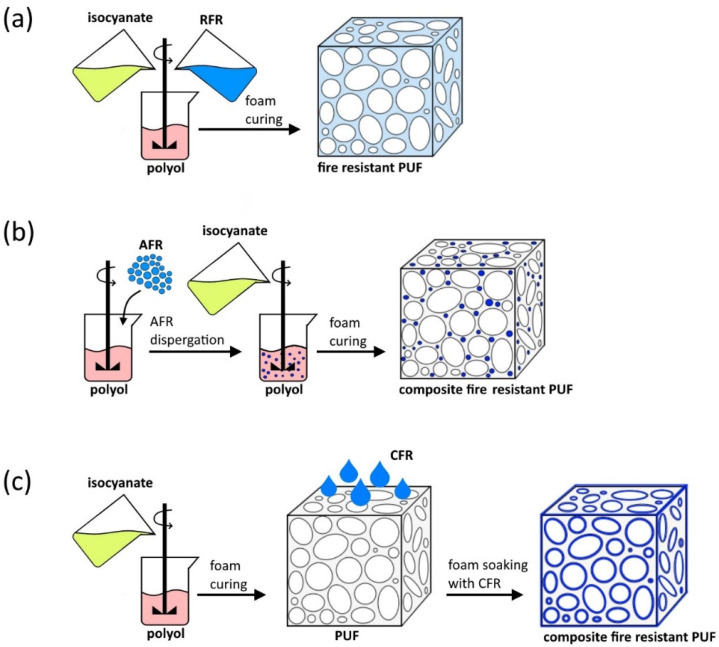
Scheme of fire-resistant PUF preparation by application of: (**a**) RFR; (**b**) AFR; (**c**) CFR.

There are multiple classifications of FRs [12,34]. For instance, classification according to the behavior of materials in connection with fire may be applied to fire-resistant PUFs [12]. According to this classification, materials can be divided into resistant to fire materials (how well materials keep their structure and mechanical properties) and fire reactive materials (how materials react to fire, e.g., hindering fire development by the elimination of one of the elements of the “fuel-heat-oxygen” cycle). FRs can also be classified according to the additive type: halogen-containing FRs, phosphorous-containing FRs, organic FRs, etc. [34]. However, classification according to the technique used to incorporate FRs into PUF structure is more widespread [35,38,40] (Figure 2). FRs are divided in three groups: reactive fire/flame retardants (RFRs), additive fire/flame retardants (AFRs), and coating fire/flame retardants (CFRs).

The RFRs form covalent bonds with starting reagents (in particular, with isocyanate) owing to the presence of various hydroxyl, amino, and epoxy functional groups. Hence, the RFRs are incorporated into the polymer chain which, on the one hand, leads to better additive dispersion ensuring uniform fire retardancy efficiency and, on the other hand, has an insignificant effect on the mechanical properties of the final product. Unlike AFRs and CFRs, the reactive FRs cannot migrate from the polymer matrix during foam exploitation and burning owing to reliable chemical bonding with the PU matrix. In addition, application of RFRs requires elaboration of new formulations of PUF production leading to an increase in the price of the final product [32,35,40].

AFRs and CFRs provide higher structural diversity and do not react with PUF ingredients. Since the modified PUF contains two or more different substances, it can be considered to be composite foam. The AFRs are usually dispersed in one of the PUF starting reagents, whereas FR coatings are formed after foam curing (see Figure 2) [38,40,43]. CFRs can be deposited on a polymer surface by employing dip-coating, layer-by-layer (LbL) assembly, sol-gel (SG) technique, etc. [41]. A layer of CFR usually acts as a physical barrier to heat and combustible gas propagation, while the function of AFRs is more complex [32,35,38,40,43]. AFR application requires comparatively high FR weight portions (more than 50 wt.% [38]) in order to significantly influence PUF fire resistance. Sometimes, such a huge content of FR leads to considerable deterioration of PUF mechanical properties and thermal conductivity [19,35,40].

Over the last two decades, considerable interest has been drawn to synthetic and natural nanomaterials as perspective environmentally friendly and sustainable components in polymer materials [34,38,46,52,53,54,55]. The PUFs modified with various nanoscale components consist of at least two phases, and thus, can be considered to be nanocomposite foams. According to many reports, nanomaterial incorporation can significantly improve PUF mechanical properties [38,40,56], thermal stability [38,52,56], sound absorption [40], and flame retardancy [31,32,38,39,46,52,56]. For instance, application of carbon nanotubes (CNTs) enhances the refractory features of PUF by reducing the rate of heat release and mass and energy transfer, as well as mechanical properties [56,57]. Importantly, the use of the nanoscale components in combination with traditional FRs can result in a synergetic effect that improves the overall flame retardancy of the final nanocomposite foam [35,38,43,52]. In summary, the application of the nanosized components is a new approach to multifunctional PUF improvement, and therefore, nanomaterials can be considered to be perspective AFRs and CFRs. Here, we review the recent progress that has been accomplished in the preparation of fireproof nanocomposite PUFs.

## 2. Fire Tests of PUFs

Ignitability, flame spread, heat, and smoke release are the main parameters that are evaluated to assess PUF flame retardancy. These parameters are usually evaluated employing a cone calorimeter test (CCT), a limiting oxygen index (LOI) test, and a UL-94 test [34,52,58].

The CCT conforms with international standards (e.g., ISO 5660) and provides insights into the behavior of polymer combustion. A sample is exposed to a conical radiant electric heater and ignited by an electric spark. The amount of released heat during sample combustion is referred to as the quantity of consumed oxygen. Parameters such as time to ignition (TTI), heat release rate (HRR), peak of heat release rate (pHRR), total heat release (THR), total smoke production (TSP), total smoke released (TSR), and smoke extinction area (SEA) can be measured using the CCT. The HRR is the most important parameter, which is usually used for an FR effectivity comparison, since it provides data on fire spread rate, fire size, and the amount of generated heat. The peak of heat release rate (pHRR) corresponds to the maximum of the HRR curve. Higher values of the HRR and/or the pHRR implicate difficult control of the fire, and hence, more considerable damage. The TTI/pHRR ratio gives the fire performance index (FPI), which can also be used for fire resistance evaluation (a polymer with higher FPI is more fire resistant). Since the danger of smoke release during real fire is comparable with that of flame, parameters such as TSP, TSR, and SEA are of the greatest importance as they give information about smoke yield [34,35,38,40,43,52,59].

Similar to the CCT, the LOI test conforms to international standards (ISO 4589) and is usually employed to study rigid polymers. Fire is applied to the top of a sample placed vertically in a glass chimney. The LOI test yields a value of the minimum oxygen concentration (in O_2_/N_2_) that is necessary to keep the sample burning for 3 min or to consume 5 cm of the sample. Hence, higher values of the LOI test correspond to better flame retardancy. If the sample does not ignite after 30 s of flame exposition, the oxygen concentration should be increased. Thus, the LOI value is an important value for assessing fire development in fire-resistant PUFs [34,35,38,40,43,52].

The UL-94 test shows if the material is prone to spread a fire or to extinguish. The bottom of a sample is ignited twice (the second ignition follows a sample extinguish after the first one) and the times of the sample’s self-extinguish are measured. Unlike the CCT and the LOI test, the UL94 test requires five parallel samples in each measurement (according to IEC 60695-11-10) [34,52].

In addition to the tests mentioned above, the thermal stability of PUFs can be studied using thermogravimetric analysis (TGA), differential thermal analysis (DTA), and differential scanning calorimetry (DSC) [35,38]. These methods provide qualitative and quantitative information on the heat absorbed/released by the foam due to phase or/and chemical transformations by indicating the rate and extent of weight loss.

## 3. Carbon Nanomaterials as Fire Retardants

Carbon nanomaterials have been extensively applied as FRs in PUFs [19,40,41,46,60,61]. The flame retardant activity of carbon materials is caused by the following factors: CO_2_ release during carbon oxidation, hindering of oxygen and combustible volatiles transmission between the polymer matrix and the external environment, significant heat isolation owing to formation of a char layer, as well as partial absorbance of smoke particles and combustible components [62,63]. FR carbon nanomaterials include carbon nanotubes (CNTs), carbon nanofibers (CNFs), graphene and its oxide (GO), and expanded graphite (EG). Here, we provide an overview of the recent progress that has been accomplished related to the application of these materials in fireproof PUFs. The corresponding CCT and LOI test results are summarized in Table 2 and Table 3.

### 3.1. Carbon Nanotubes (CNTs)

CNTs are carbon allotropes which have a cylindrical shape and consist of one or several concentric graphite layers. The remarkable structural and electronic properties as well as the outstanding mechanical characteristics of CNTs (e.g., high aspect ratio, strength, and stiffness) have attracted particular attention in the development of PUF nanocomposites [61,64,65,66].

However, recent reviews of the literature have revealed that the application of CNTs as fireproof components or as a component of an FR system is rather rare compared to other carbon nanomaterials [40,61]. Since pure nanotubes cannot provide proper flame retardancy of the final nanocomposites, CNTs are usually applied as a component of CFRs. Taking into account the negative charge of CNT surface and a great number of CNT surface modification approaches, the LbL technique seems to be the best option for PUF modification [57,67,68,69]. It should be noted that the LbL technique can create multilayer coatings on a charged substrate which is alternatively immersed into positively and negatively charged species. The electrostatic interaction between oppositely charged layers is the main driving force of this procedure; however, other interactions (e.g., donor/acceptor [70,71], hydrogen bonding [72,73], or covalent bonding [74,75]) can also be employed. Thus, the LbL technique is applicable for assembling a variety of materials such as polyelectrolytes, nanoparticles, or biomolecules [62].

Thus, for instance, aqueous CNT suspensions stabilized by using polyacrylic acid (PAA) and montmorillonite (MMT) were utilized as a component of bilayer (BL) coatings of flexible PUFs in [67,68]. In some cases, CNTs are modified/grafted for better interaction with other layers [57,69]. For example, CNTs modified by using branched polyethylenimine (PEI) had a better interaction with the PAA layer [69], whereas chitosan (CS) grafted CNTs, MMT, and alginate formed a trilayer (TL) structure [57].

The fire tests performed in the abovementioned studies revealed significant amelioration of the fireproof properties of the final composite foams. Fire tests demonstrated a tremendous reduction in the pHRR value of the final composite foam in [69]. Reductions in the pHRR and TSR values of 67% and 80%, respectively, were observed in the case of composite 6BL PUF and immediate self-extinguishing after flame removal in the case of 9BL PUF [67], whereas deposition of 20BL of polyaniline and MMT-stabilized CNTs led reductions in the pHRR, THR and TSR values of 51%, 37% and 47%, respectively [68]. The 4TL and 8TL PUFs revealed a dramatic reduction in the pHRR value (up to 69%) [57].

However, the application of CNTs is not limited to only the LbL technique. To provide better heat transfer of organic phase-change material (capric acid) encapsulated in PMMA capsules, the latter has been modified with CNTs and Fe_3_O_4_ nanoparticles [76]. In addition to heat transfer function, the CNT/Fe_3_O_4_ component provided enhanced fire resistance of rigid PUF after its modification with obtained thermoregulating nanocomposite.

CNTs are not the only type of 1D carbon nanomaterials that can be employed as FRs in PUFs. For example, carbon nanofibers (CNFs) have been employed as AFR [77] and CFR [78] additives. Incorporation of CNFs into the polymer matrix led to a 35% reduction in the pHRR value compared to a control sample [77], while 4 BL coating consisting of CNF/PEI and PAA layers provided significant reductions in the pHRR and THR values (40% and 21%, respectively) and prominent burning time reduction (21%) [78]. Interestingly, the smaller CNF mass fraction in CFR (1.6% [78]) provided better fire protection than the larger CNF mass fraction (4%) embedded in the polymer matrix in a study by M. Zammarano et al. [77].

Summing up, CNTs and CNFs promote higher thermal stability and improved formation of the char layer, which acts as a physical barrier to heat and flammable gas propagation. Obviously, the thickness of CFR plays a considerable role in PUF fire resistance, i.e., the thicker CFR layer results in better fireproof properties. The synergic effect of CNTs and MMT should be underlined, since MMT provides additional heat/gas diffusion hindering, while CNTs make the layer structure thicker, more uniform, and hence, more reliable [57]. Some fire retardant properties of the reviewed CNT/CNF-based FRs are collected Table 2.

### 3.2. Graphene and Graphene Oxide

Graphene is another carbon allotrope which consists of a single layer of atoms arranged in a hexagonal lattice nanostructure [79]. Similar to CNTs, pristine graphene cannot provide the appropriate flame retardancy level for PUFs [80], hence, the common strategy is to apply it as a synergetic component in CFRs to enhance the effect of conventional FRs [61,62]. For instance, graphene nanoplatelets were applied in combination with nickel (II) oxide as AFR to improve fire retardancy of rigid PUF [80]. The fireproof components were added via an in situ polymerization method which led to an increase in foam density and enhancement of the mechanical properties (Young’s modulus, tensile strength, and elongation at break). According to the fire tests, graphene provided a barrier effect, helping to prevent material consumption and flame diffusion, whereas NiO catalyzed CO oxidation to inflammable CO_2_. This synergetic effect resulted in an LOI increase to 30.5% and self-extinguished a sample containing only 2 wt.% of NiO and 1.5 wt.% of graphene.

**Figure 3:**
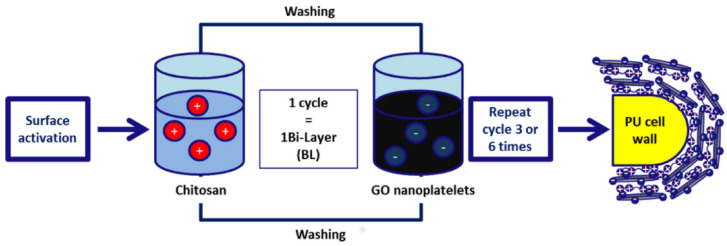
A scheme demonstrating the LbL-assembled fire-retardant nanocomposite PUF. Reproduced from [87].

Graphene oxide (GO) has wider applications as an FR than graphene since it has many oxygen-containing functional groups (e.g., OH, COOH, and epoxy group). GO particles in aqueous suspensions exhibit a negative charge, making GO an attractive nanomaterial for the LbL technique [81]. As a result, recently, GO has become a component of various multilayer CFRs [82,83,84,85,86,87,88,89,90,91,92,93]. The LbL technique requires PUF surface activation (positive or negative charging), which is usually achieved by treatment with diluted inorganic acids (e.g., HNO_3_ [94]) or/and PAA) [87]. Surface activation is followed by consecutive PUF dipping/immersion to either positively charged polymer solution (e.g., chitosan, dopamine) or negatively charged particle suspension (GO aqueous dispersion). Depending on the number of compounds employed, bilayer (BL), trilayer (TL), or even quadlayer (QL) coatings can be obtained. GO can be used along with other nanosized objects, for example, with β-FeOOH nanorods [83] and amino-terminated silica nanospheres (KH-550-SiO_2_) [95]. An example of the typical BL deposition on PUF is presented in Figure 3.

The stability of GO dispersion has considerable importance for GO-based CFRs. A possible GO coagulation may result in non-uniform distribution of FR in/onto PUF; hence, aqueous GO solutions are often stabilized, for instance, by sodium alginate (SA) [95].

Importantly, the LbL technique is not the only way to deposit GO-containing CFR. As an alternative, the electrostatic interaction of negatively charged GO with positively charged strong polyelectrolyte poly(diallyldimethylammonium chloride) (PDAC) was employed by Carosio et al. to coat the complete surface of a polymer matrix by using PDAC/GO exoskeleton comprising highly oriented GO nanoplatelets [86]. The 3BL coating suppressed flame spread and completely prevented foam ignition, whereas the 6BL coating withstood the penetration of a flame torch. In another study, FR nanocoating was obtained by oxidative polymerization of dopamine monomer within an aqueous liquid crystalline GO scaffold [85]. The PDA/GO nanocoatings were applied to PUF and significantly improved its fire resistance: a 65% reduction in the pHRR value at 5 wt.% PDA/GO loading in an 80 nm thick coating.

GO reduction (e.g., reduction of carboxyl groups) can significantly influence the interaction of the latter with other components of CFR [82,93]. Reduced GO (rGO) has better thermal stability than GO, and was employed in [82] to obtain fireproof PUF. The combination of both carbon nanomaterials can achieve high thermal stability owing to rGO and excellent fireproof properties owing to GO.

**Figure 4:**
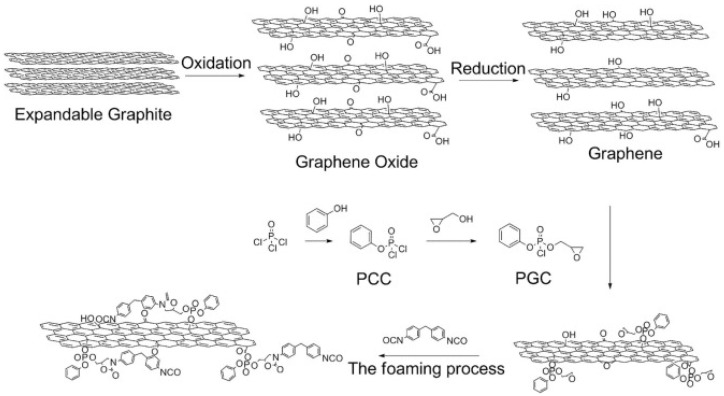
A scheme demonstrating GO reduction/functionalization. Reproduced from [97].

In addition to GO reduction, GO can be further functionalized via -OH and -COOH groups of various compounds which considerably vary its application in CFRs [94,95,96,97,98,99,100]. An example of GO reduction and subsequent functionalization is presented in Figure 4.

Functionalized GO (fGO) is usually grafted by using FR compounds, which provide certain additional fire retardant properties to the final product. For instance, nanocomposite PUF containing GO functionalized with 3-aminopropyltriethoxysilane and boric acid revealed stronger fireproof properties (pHRR of 182.2 kW/m^2^) than that produced using non-functionalized GO (pHRR of 186 kW/m^2^) [96]. In another work, the addition of fGO, obtained similarly to [96], to PUF led to an increase in the LOI value (from 27.5% to 28.1%) compared to PUF with GO [98]. Very recently, ionic liquid ([BMIM]PF_6_) functionalized graphene oxide (ILGO) was synthesized and added to flexible PUF along with other FRs [100]. The LOI and pHRR values of PUFs with ILGO and GO were 29.0% and 28.2%, and 98.56 and 130.77 kW/m^2^, respectively. In these studies, the fGO showed better thermal stability and better flame retardance due to the presence of either boron or phosphorous compounds and catalyzation of char yield resulting in better barrier effect. Moreover, the amelioration of the mechanical properties of fGO-modified composites should be neglected.

**Figure 5:**
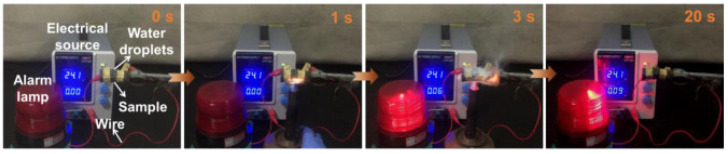
Demo flame detection process of PU-APP@GO-F after being exposed in a heavy rain, showing rapid flame detecting response. Reproduced from [102].

The fire alarm function is another fascinating property of GO-containing FRs, which, recently, has been extensively studied [101,102,103,104,105,106,107]. For example, flexible PUF was dip-coated using APP-modified GO [102]. The deposited CFR was reinforced using fluorine-containing silane for better fire retardancy. During burning, APP and silane decomposed, releasing elemental P, Si, and F, and thus, quenching radicals’ diffusion, meanwhile GO acted as physical barrier and provided temperature-responsive resistance. The flame detection response time was only 2 s, whereas the fire early warning time in pre-combustion was 11.2 s at 300 °C (see Figure 5). A multifunctional fire-resistant and fire-sensitive nanocomposite PUF/PVH/PA@GO/CNTs@PVH/PA/BN was obtained recently via the LbL technique [107]. The primer layer containing flame retardant copolymer (PVH) modified by phytic acid (PA) improved the PUF’s surface flatness and flame retardancy. The middle layer composed of GO/CNTs served as a fire sensor to detect the temperature change. The surface layer of PVH/PA/boron nitride (BN) coating worked as a shield to protect the underlying sensor and promoted its temperature-response performance. Such a multilayer CFR provided outstanding fireproof properties of the final PUF nanocomposite: the LOI increased from 18 to 58% and the pHRR and THR values were reduced by 49% and 33%, respectively.

Summing up, the fireproof properties of graphene and its oxide are very close to those of CNTs. These carbon nanomaterials possess good thermal stability and outstanding mechanical properties and they are prone to form char layer which acts as a physical barrier to gas and heat diffusion via its high specific surface area. In combination with other FRs (e.g., phosphorous/nitrogen containing), graphene and GO improve the flame retardant property and smoke suppression effect of foam. The Table 2 summarizes some fire retardant properties of the reviewed graphene/GO-based FRs.

**Table 2:** Results of the CCT and the LOI test of various CNT- and graphene/GO-containing composite PUFs.

FR	FR Type	LOI (%)	Δ pHRR (%)	Δ THR (%)	Δ TSR (%)	Ref.
MMT/CS-CNT/SA (8TL)	CFR	–	−78	−3	–	[57]
PAA/CNT-PEI/PEI (4TL)	CFR	–	−35	−21	–	[69]
PEI-Py/PAA + CNT (6BL)	CFR	–	−68	−3	−78	[67]
PEI-Py + CNT/PAA (6BL)		–	−68	−4	−76	
PEI-Py + CNT/PAA + CNT (6BL)		–	−67	−9	−80	
PANi/CNTs-MMT (20BL)	CFR	–	−57	−37	−47	[68]
Graphene/NiO	AFR	31	–	–	–	[80]
GO/PEI	CFR	–	−73	+18	−57	[82]
rGO/PEI		–	−65	+7	−14	
GO/FeOOH (5BL)	CFR	–	−50	+7	–	[83]
GO/CS/AL (10TL)	CFR	–	−60	–	−31	[84]
PDA/GO	CFR	–	−65	−12	–	[85]
GO/PDAC (3BL)	CFR	–	−60	–	–	[86]
GO/CS	CFR	–	−54	–	−59	[87]
GO-SiRF	CFR	32	−64	−35	–	[88]
GO/CS (5BL)	CFR	–	−46	−13	–	[90]
GO_A_/CS (6BL)	CFR	–	−54	−10	−76	[91]
GO/SiR	CFR	30	–	–	–	[92]
PEI/APP-rGO	CFR	–	−64	−23	–	[93]
E bbPAA/PEI/PDA-rGO 6TL	CFR	–	−35	+39	−52	[94]
GO/KH−550-SiO_2_	CFR	–	−51	–	–	[95]
fGO/EG/DMMP	AFR	–	−33	−25	–	[96]
PUF-fGN	AFR	–	−56	−45	–	[97]
fGO/EG/DMMP	AFR	28	–	–	–	[98]
EG/APP/ILGO	AFR	29	−71	−56	–	[100]
SiP/GO/PFDTS	CFR, alarm	–	−78	–	–	[101]
rGO–SiR	CFR	32	−65	–	−30	[103]
GO-NR/MMT/PEG	CFR, alarm	–	–	–	–	[104]
GO/HCPA (4BL)	CFR, alarm	37	−60	−35	–	[105]
GO@HPTCP/CNT (15BL)	CFR, alarm	29	−63	–	–	[106]
PVH/PA@GO/CNTs@PVH/PA/BN	CFR, alarm	58	−49	−33	−42	[107]

### 3.3. Expanded Graphite

Expandable graphite (EG) is one of the most efficient FRs for PUFs. It is synthesized during graphite intercalation with various acids (sulfuric, acetic, or nitric acids) [108]. The exposition of EG to high temperatures (>170 °C) results in tremendous volume expansion (~50–250 times [46]) because of simultaneous carbon oxidation and acid thermal decomposition followed by release of various gases (e.g., CO_2_, SO_2_, NO_2_, etc.). Thus, EG can be considered to be a low cost one-component intumescent, implying that it combines an acid source, carbon source, and blowing agent [109].

As well as other carbon-based nanomaterials, EG is usually applied as and AFR in combination with other FRs, which leads to remarkable improvement of the overall fire retardant properties of nanocomposite PUFs [110,111,112,113,114,115,116,117,118,119,120,121,122,123,124,125,126,127,128,129,130,131,132,133,134,135,136,137,138,139,140,141,142]. Recently, the influence of EG particle size on fireproof properties has been investigated [117,118]. The 10 wt.% loading of EG particles with an average size of 300 µm and 500 µm led to an increase in the LOI of 29.8% and 31.8%, respectively, indicating that bigger EG flakes provide better fire retardancy. One of the possible explanations of these phenomena is that EG forms an interconnected structure in a foam matrix. Nevertheless, a usual high EG loading level [46] deteriorates some mechanical (e.g., decreased compressive strength) and isolating (e.g., higher foam density and increased open cell number lead to worse thermal conductivity) properties [40]. In particular cases, EG has been added as CFR [143,144]. Thus, S. Wang et al. [143] used a mixture of silicone resin (poly-DDPM) and EG to brush rigid PUFs. In addition to a significant increase in the LOI value (from 18% to 32.3%) and decreases in the PHR (by 55%) and the peak smoke release rate (by 59%), the compressive strength of the coated PUF was impressively increased (by 10%).

EG interfacial compatibility with the polymer matrix can be improved by using EG chemical modification/grafting (e.g., via epoxide and carboxylate groups [40,46]). As a result, EG links via hydrogen bonding with amino groups of such compounds as chitosan. Recently, this bonding was employed to coat flexible PUF using the dip-coating method [144]. In another work, EG was successfully bonded with SA to be used as an effective CFR [145]. Encapsulation of EG is an alternative strategy for interfacial compatibility improvement. For instance, EG encapsulation in magnesium hydroxide (MH) led to better interface adherence and considerably enhanced flame retardance of nanocomposite foam (LOI of 32.6%) [146]. Nevertheless, although interest in nanocomposite PUFs based on modified EG has been continuously growing, the number of studies on EG chemical modification is still rather limited [145,146,147,148,149,150,151,152].

**Figure 6:**
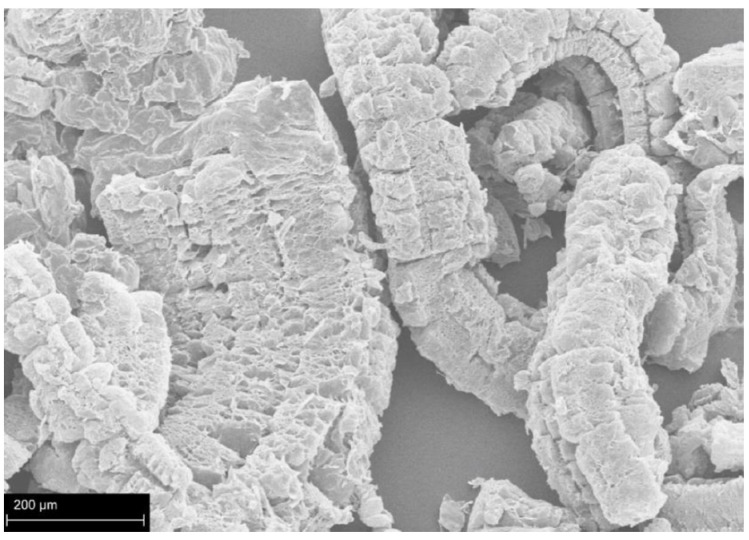
SEM micrograph of EG’s worm-like structure. Reproduced from [137].

As noted above, EG is considered to be intumescent. A thermally induced EG volume expansion is followed by formation of char worm-like structures (see Figure 6) that impede heat/flame transfer and flammable gases diffusion, hence, reducing material’s temperature. Moreover, EG expansion is usually accompanied by release of nonflammable gases (e.g., SO_2_, NO_2_, and CO_2_) which dilute/displace flammable gases and lead to self-extinguishing [40,46,61]. Some fire retardant properties of the metioned above EG-based FR are presented in Table 3.

**Table 3:** Results of the CCT and the LOI test of various EG-containing composite PUFs.

FR	FR Type	LOI (%)	Δ pHRR (%)	Δ THR (%)	Δ TSR (%)	Ref.
5ADPO_2_/10EG	AFR	–	−63	–	–	[130]
EG	AFR	32	−54	−47	−84	[117]
EG	AFR	30	−53	−40	−80	[118]
EG	AFR	21	−36	−22	−48	[119]
EG	AFR	22	−61	−43	−83	[132]
EG30	AFR	55	−74	–	–	[139]
EG/Borax	AFR	27	−84	−63	–	[110]
EG/APP	AFR	30	–	–	–	[112]
EG/APP	AFR	29	−58	−43	–	[124]
EG/DTP	AFR	30	−40	−12	–	[114]
EG/MCC	AFR	25	–	–	–	[129]
EG/DDP	AFR	28	−83	−40	–	[142]
EG/PDEO	AFR	–	−51	−51	–	[115]
EG/Cloisite	AFR	29	–	–	–	[122]
EG/BDMPP	AFR	22	−58	−48	−48	[136]
EG/PDEP	AFR	27	−57	−24	−29	[134]
EMD8-EG	AFR	31	−56	−42	−46	[113]
EG/SiO_2_/[emim] [BF4]	AFR	–	−70	−19	−57	[128]
EG/MP	AFR	–	−25	−24	−6	[127]
EG/Mpi		–	−16	0	+15	
EG/MITS	AFR	25	−62	−8	−78	[141]
EG/AHP	AFR	26	−26	−14	–	[138]
EG/Zr-AMP	AFR	31	−74	−61	–	[120]
EG/ATH/BH	AFR	34	−64	−26	−45	[123]
ADP10/EG20	AFR	26	−4	+24	–	[133]
PMCP/EG	AFR	27	−43	−24	–	[140]
TGD/DMMP/EG-ATH	AFR	33	−68	−74	−7	[121]
EG/Phenylphosphonic-aniline salt	AFR	30	−45	−24	−58	[125]
EG/APB	AFR	28	−58	−43	–	[126]
EG/PEPA	AFR	32	−65	−37	−74	[116]
EG/DOPO	AFR	30	–	−27	−16	[135]
EG/BDEMPP	AFR	26	−45	−36	–	[131]
EG/Si-resin	CFR	32	−55	−22	–	[143]
EG/CS	CFR	31	−87	−87	−98	[144]
EG/ADPO2/SA	AFR	26	−23	–	–	[145]
EG@MH	AFR	33	–	–	–	[146]
EG@ATH	AFR	30	−8	–	–	[147]
EG*_x_*/APP	AFR	30	−54	−14	–	[148]
PUEG/GMAAPP	AFR	25	–	–	–	[149]
EG-MCA	AFR	29	−61	–	–	[150]
IL-EG/DPES	AFR	–	−54	−36	−65	[151]
EG-silane (KH550)	AFR	32	–	–	–	[152]

## 4. Nanoclay Fire Retardants

**Figure 7:**
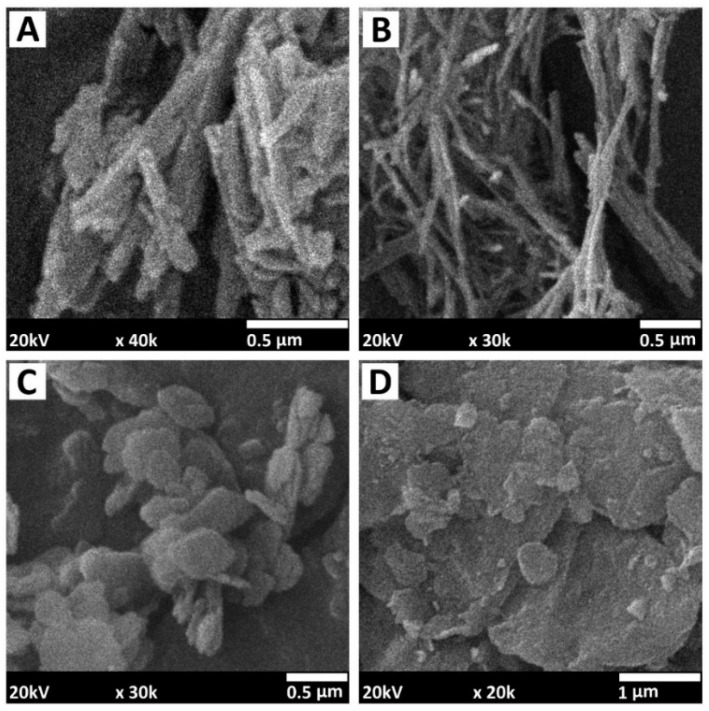
SEM micrographs of: (**A**) halloysite, (**B**) sepiolite; (**C**) kaolinite; (**D**) montmorillonite. The images are collected with a JEOL JIB 4501 multibeam system at accelerating voltage of 20 kV and spot size 15.

Nanoclays (NCs) are nanosized silicates (e.g., aluminosilicates, magnesium silicates, etc.) that can be extracted from soil. Owing to the fortunate combination of low price because of natural abundance and outstanding properties (e.g., mechanical and thermal stability), recently, nanoclays have attracted considerable interest and have become popular components of polymer nanocomposites [153,154,155,156,157]. Obviously, the is an enormous variety of clays and there are many classifications of these materials. Here, NCs are divided in two groups: one-dimensional and two-dimensional nanomaterials. Thus, for example, halloysite and sepiolite can be referred to as one-dimensional nanomaterials, while hydrotalcite, kaolinite, and montmorillonite belong to the group of two-dimensional nanomaterials (see Figure 7).

### 4.1. One-Dimensional Nanoclays

Halloysite (or halloysite nanotubes (HNTs)) is a tubular (1D) aluminosilicate with the chemical formula Al_2_(OH)_4_Si_2_O_5_·4H_2_O. Aluminosilicate nanosheets are rolled in such a way that the HNT exterior surface has a conner-shared tetrahedral SiO_4_ layer, and thus, has a negative charge, whereas the nanotube lumen surface consists of an edge-shared octahedral AlO_6_ layer and is positively charged [158]. Sepiolite nanofibers have a layer of magnesium ions with octahedral coordination and two layers of silica in a tetrahedron; the chemical formula of sepiolite is Mg_4_Si_6_O_15_(OH,F)_2_·6H_2_O. As well as HNTs, sepiolite fibers are negatively charged [159]. The outer surface of both NCs has the silanol groups (Si-OH) and can be chemically modified/grafted which can result in a change oin the surface charge. Due to outstanding mechanical properties, high aspect ratio, surface area, and thermal stability, as well as negatively charged surface, and the possibility of its modification, one-dimensional NCs are considered to be affordable replacements for more expensive CNTs in various PU nanocomposites [52,160].

**Figure 8:**
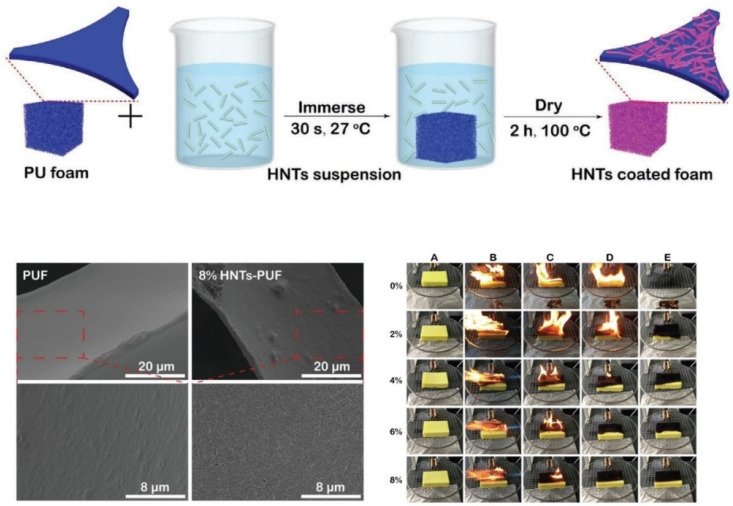
Dip-coating process of flexible PUFs in aqueous HNT solution, SEM micrographs of non-coated and coated PUFs, fire resistance tests. Reproduced from [165].

For instance, an SA-stabilized sepiolite aqueous solution and PEI were deposited on flexible PUF using the LbL technique [161]. The deposition of 6 BL (SA-sepiolite/PEI) led to a reduction in the pHRR and THR values from 710 to 170 kW/m^2^ and from 32.6 to 24.8 MJ/m^2^, respectively. Smoke production was also considerably reduced (e.g., total smoke production reduction by 25%) owing to the formation of a uniform thick thermally resistant char/sepiolite layer impending gas diffusion. During the last 5 years, HNTs have frequently been applied for improvement of PUF fire resistance [162,163,164,165,166]. F. Wu et al. [165] treated flexible PUF with an aqueous solution of HNTs using the dip-coating technique (Figure 8). The introduction of HNTs transferred the foam surfaces from hydrophobic to super-hydrophilic (contact angle decreases from 116° to 0° after HNT coating), improving thermal stability and fire resistance. The same flame retardant effect was observed in composite PUF after its coating with PEI/APP/HNT film [166]. The foam primarily activated with PAA was immersed in 4 wt.% PEI—8 wt.% APP—10 wt.% HNT solution for 1 min. Such an express coating procedure was enough to provide a reduction in the pHRR and THR values of 52.5% and 3%, respectively. It is noteworthy that the pristine foam and foam treated only with the PEI-APP solution displayed almost the same fireproof properties, indicating the key role of HNTs in the flame retardancy enhancement of the final composite. The presence of HNTs slows down fire spread via formation of a more stable char protection which preserves foam from further degradation.

Despite rather promising results demonstrated by nanocomposite PUFs treated with HNTs, in fire tests, flame retardancy can be increased with the help of chemical modification of the halloysite surface. For example, the HNT surface can be grafted by HDTMS and TEOS, giving polysiloxane-modified HNTs (POS@HNT) [164] or modified with branched PEI (BPEI), as has been accomplished by R.J. Smith et al. [163]. In addition to outstanding torch flame resistance during 10 s, the PUF coated by using POS@HNTs exhibited super-hydrophobicity, and therefore, it was possible to use nanocomposite PUFs for efficient and recyclable oil absorption [164]. At the same time, the LbL deposition of PAA-stabilized HNTs and BPEI-HNTs on the PUF surface resulted in a tremendous enhancement of fire retardance: CFR consisting of 5 BL reduced the pHRR value by 62% and the TSR value by 60%. HNTs act as barriers for mass and heat transfer, hence, significantly delaying flame spread and preventing melt dripping, without collapsing the foam structure [163].

The application of one-dimensional NCs as fire retardant components of composite PUFs is very similar to that of CNTs. NCs are rarely employed as a single FR component, being a synergist component of multicomponent FR. The negative surface charge and possibility of surface modification prompts the use of NCs as a component in CFR systems (e.g., LbL deposition). As a component of a CFR system, one-dimensional NCs can be referred to as non-intumescent agents that provide a shielding effect during polymer thermal decomposition, which slows down the transfer of heat, oxygen, mass, and volatile products. Remarkable suppression of smoke production can also be linked to formation of a uniform and stable barrier that impends further gas diffusion as well as water release from NC structure at elevated temperatures.

### 4.2. Two-Dimensional Nanoclays

The group of two-dimensional NCs includes a number of materials: montmorillonite (MMT), kaolinite, vermiculite (VMT), bentonite, dellite, laponite, mica, and hydrotalcite (HT). Among these clays, MMT has attracted the interest of researchers as a promising natural FR additive. MMT is 2:1 clay, implying that two sheets composed of SiO_4_ tetrahedra lay on either side of the sheet containing AlO_6_/MgO_6_ octahedra [167]. VMT has the same structure as MMT; however, it is a hydrated silicate mineral that expands on heating. Kaolinite has the same chemical formula as halloysite, nonetheless, unlike the latter, it is presented by 2D hexagonal crystals consisting of stacked layers [168]. The neighboring aluminosilicate layers in NCs are linked via van der Waals forces. The interlayer distances in NCs considerably vary from one material to another. For example, the widths of MMT and kaolinite interlayer spaces are 1.23 nm and 0.71 nm, respectively. The interlayer space of NCs can be intercalated by small molecules. Thus, the ultrasonic treatment of NC fillers in PU components (e.g., polyol) may lead to clay intercalation or even exfoliation, which finally results in better clay dispersion, and hence, more effective heat barrier effect [59]. Unlike the previously described NCs, HT refers to a layered double hydroxide (LDH) group. The bivalent and trivalent cations (usually presented by Mg^2+^ and Al^3+^) are octahedrally coordinated to six hydroxyl groups and form a positively charged layer. The HT interlayer space contains water molecules and anions (usually presented by CO_3_^2−^) which compensate for the positive charge. As well as other NCs, the interlayer space of HT can be modified by various inorganic and organic anions [169].

Layered NCs can be used as individual FRs. For instance, A. Agrawal et al. added kaolinite to polyol as AFR [170]. An aqueous solution of NC was treated using 1% APTES solution in order to improve the adhesion between the matrix and filler. The results of TGA and fire tests indicated enhancement of thermal stability and fireproof properties (reductions in pHRR, THR and TSR of 25%, 29% and 65% respectively) of nanocomposite foams. However, as well as other nano-sized components of composite PUFs reviewed above, layered NCs are usually applied in combination with other FRs. Thus, VMT/CS 8BL structure significantly reduced the pHRR and TSR values of flexible PUF by 53% and 63%, respectively [171]. High-aspect-ratio mica stabilized by PAA and CS have been used as components of BL coating for PUF [172]. The composite foam with 8 BL withstood a 10 s torch flame test and self-extinguished. The pHRR and TSR values were reduced by 54 and 76%, respectively, owing to reinforcement of the protective char layer by the mica particles. Such a reinforced char layer provides a better barrier effect, suppressing smoke release and impeding flammable gases and heat diffusion.

In another study, cationic starch and MMT were used as components of CFR introduced by using spray coating [173]. Despite the fact that the 5 BL structure did not inhibit flame propagation, it successfully prevented the melt from dripping and preserved the inner part of the foam. The pHRR and THR values were reduced by 22.7% and 52.7%, respectively. MMT can also become a component of more complex CFR systems. Thus, in [174], the PUF surface was firstly modified using alginate-stabilized MMT/CS/poly-D-lysine TL, and then, coated with CS-PA intumescent layer. Various flame resistance tests revealed outstanding improvement of nanocomposite PUF due to the synergetic effect of TL structure providing higher thermal stability and a more uniform coating and intumescent agent ensuring production of char isolating barrier. In some FR systems, MMT is not the only nano-sized component and it is used in combination with CNTs [57,68], GO [104], sepiolite [175] and other materials [176]. For instance, the modification of salvia filler with MMT particles considerably improved interphase compatibility between filler and the polymer matrix. This fact led to better filler dispersibility which finally resulted in well-developed foam structure and improved mechanical, thermal, and flame-retardant performances. Earlier various multilayer structures (BL, TL, and QL) including BPEI, PAA, sodium montmorillonite (Na-MMT), and LDH were deposited on the flexible PUF using the LbL technique [177]. The nanocomposite foam with the PAA/LDH/BPEI/Na-MMT quadlayer revealed a decrease in the pHRR value of 31% and a decrease in the THR value of 21%. The LbL approach was also employed very recently by S. Abrishamkar et al. [178] to coat flexible PUF using LDH and modified GO layers. The obtained composites demonstrated good mechanical properties, increased char yield, and outstanding improvement of the fire safety properties. Although the enhanced flame retardance was primarily caused by GO modification, it should be noted that LDH presence in the BL structure provided better interaction with modified GO, resulting in thicker and denser coating on the foam surface. In the work of H.-K. Peng et al. [179], non-modified HT mixed with phosphorous-based FR (FR-047) was used as AFR filler in rigid PUFs. Foam modification provided an increase in the LOI value of 4%. Again, the layered NC-reinforced char layer providing better barrier functions.

**Figure 9:**
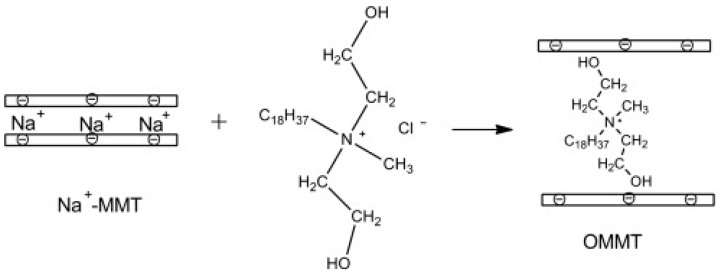
A scheme of the preparation of the organically modified montmorillonite (OMMT). Reproduced from [180].

**Figure 10:**
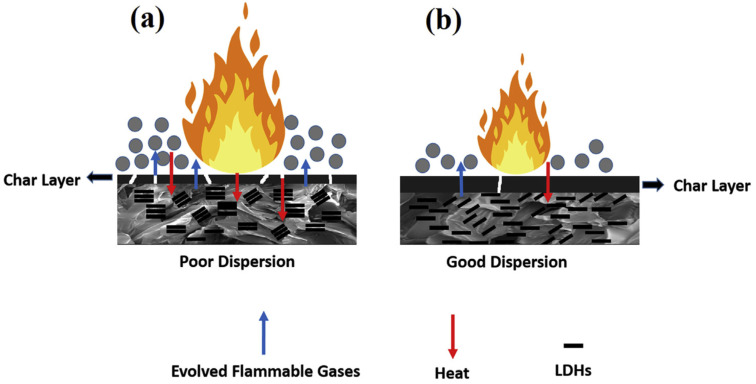
The influence of chemical modification of the layered NC on its dispersion in the polymer matrix and the final flame retardant properties: (**a**) The non-modified layered NC agglomerate in the polymer matrix, leading to the poorer char formation, and thus, worse flame retardant properties; (**b**) the exfoliated, during chemical modification, layered NC is well dispersed and provides better flame retardances through hindering of the heat and the flammable gases diffusion. Reproduced from [182].

As mentioned above, the chemical modification of the interlayer space in layered NCs provides further improvement of fire retardant properties of clay-based FRs [180,181,182,183]. The chemical functionalization implies intercalation or even exfoliation of the layered NC (Figure 9). In the work of X. Zheng et al. [180], organically-modified montmorillonite (OMMT) mixed with phosphorous-containing APP and TPP was introduced as AFR in flexible PUF. Recently, OMMT was further functionalized with phosphorus-containing organosilicon compound (PCOC) which led to a significant increase in interlayer space and NC exfoliation [181]. According to the CCT results, additional functionalization of OMMT decreased the pHRR and TSR values by 51% and 40%, respectively, compared to neat foam and by 47% and 37%, respectively, compared to PUF/APP/OMMT. Such a significant difference between the CCT results for functionalized and non-functionalized OMMT is believed to be due to the higher degree of clay intercalation/exfoliation, hence, better clay distribution in the PU matrix, and thus, promotion of a denser and more uniform char layer. Phosphorous-containing agent was also introduced in HT interlayer space and the obtained composite was applied as AFR to flexible PUFs [183]. The provided CCT and LOI test revealed dramatic improvement in the fire resistance of composite foams, which can be explained through the synergetic effect of LDH and phosphoric acid, i.e., the crystal water contains LDH and absorbs the heat, and hence, lowers the temperature, meanwhile phosphoric acid promotes the carbonization reaction and oxygen isolation. The interlayer space of LDH has also been modified with sulfonate-containing calix[4]arenes [182]. Again, the obtained intercalated clay was employed as AFR filler for castor oil-based flexible PUFs. The authors noted that application of calix[4]arenes macrocycles improved LDH dispersion quality in the polymer matrix (as well as in [181]), which led to a higher char yield, and finally resulted in good smoke suppression and flame retardancy (Figure 10).

Taking into account the recent studies on the use of layered 2D NCs as FR additives in PUFs, one can note their multifunctionality. In addition to enhancement of the mechanical and thermal stability of the final composite, and hence, retardation of PUF thermal decomposition and release of flammable gases, the layered NCs can be intercalated/modified by various organic and inorganic substances which can act as additional FR agents. Thus, unlike one-dimensional NCs, two-dimensional NCs can be considered to be both intumescent and non-intumescent FR additives. The two-dimensional NCs used individually or as a component of multicomponent CFR systems revealed remarkable fireproof activity owing to the incorporation in the char barrier layer, thus, enhancing suppression of the gas/heat diffusion and retarding flame propagation. Some fire retardant properties of the reviewed above clay-based FRs are collected in Table 4.

## 5. Other Nanosized Fire Retardants

**Figure 11:**
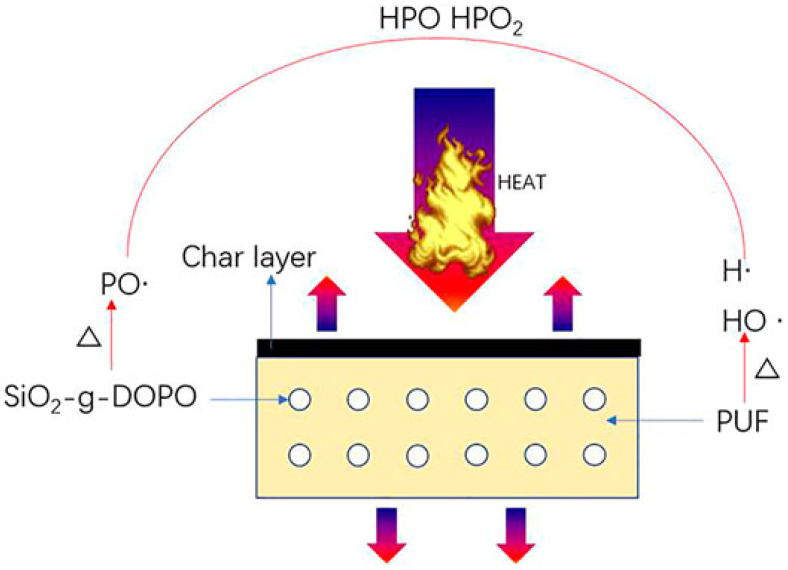
The mechanism of the synergetic effect of flame retardancy in nanocomposite PUF. Reproduced from [191].

Obviously, the carbon nanomaterials and nanoclays mentioned above are not the only nanosized FR additives applied in composite fireproof PUFs. For instance, thermal stability and flame retardance of PUF have been improved after incorporation of basalt wastes [184]. Very recently, fly ash was employed to enhance fire resistance of PUFs [185,186]. It is noted that the incorporation of basalt wastes and fly ash in the PU matrix led to better thermal stability of the composite foam (hence, higher temperatures of PU thermal decomposition) and higher char yield. Inorganic nanomaterials such as nanoparticles (NPs) are another class of FR additives occasionally used in composite PUFs [120,187,188,189,190,191,192,193,194]. For example, silica NPs have been introduced in PUFs via sol-gel deposition [187,189]. In both studies, the PUFs were immersed in water solutions containing TEOS, ammonia solution, and alcohol. At the end of the reaction, TEOS transformed to silica gel, whereas freeze-drying in [189] turned it onto aerogel. Upon combustion, silica nanoparticles prevented dripping of flaming residues (observed for non-treated foams) via increasing polymer viscosity and formed a compact and stable silica-rich hybrid char layer. The barrier effect of the latter led to a significant decrease in the pHRR value (55% [187] and 40% [189]) and the THR value (21% [187] and 29% [189]) and inhibited the release of smoke and combustible gases. Earlier, a remarkable reduction in the pHRR value of 80% was observed for flexible PUF treated with alumina aerogel [194]. As well as other nanomaterials, NPs can be carriers of various FR additives. Composite AFRs were prepared by DOPO immobilization on the surface of silica aerogel [190] and TiO_2_ NPs [188]. In these works, a dramatic increase in the fire resistance of final composite foams was achieved owing to the synergetic effect of nanoscale carriers and phosphorous-containing FR (Figure 11). Metal oxide NPs were applied in composite PUFs very recently [191,192,193]. The MgO and ZnO NPs added in combination with ATH increased the LOI values of the corresponding PUFs by 3% [192], whereas modification by CuO-loaded Fe_3_O_4_@ZIF-8 nanocomposite (where ZIF-8 is zeolitic imidazolate framework) decreased both magnetic properties and the pHRR value by 69% [193]. In addition to NPs, various two-dimensional inorganic nanomaterials have been tested as promising FRs. For instance, MXene (layered Ti_3_C_2_)/CS BLs were deposited on flexible PUF via the LbL technique [195]. According to an analysis of the burned samples, MXene nanosheets formed thermally stable flakes under elevated temperatures. Moreover, the formation of TiO_2_ was observed, implying a decrease in the oxygen concentration in this zone. Newly formed TiO_2_ could also serve as a catalyst of cross-linking and charring of CS and PU, thus, additionally improving fire safety performance.

Summing up this section, we conclude that various inorganic nanomaterials (e.g., NPs, layered structures) are basically employed as non-intumescent FRs that provide anti-dripping and barrier effect owing to the formation of a stable hybrid char layer. However, modification or a combination of nanomaterials with various compounds (e.g., organic phosphorus-/nitrogen-containing FRs) could result in the possibility of further enhancement of PUF fireproof properties owing to the synergetic effect. Some fire retardant properties of the reviewed materials are collected in Table 5.

## 6. Key Challenges and Future Opportunities

Nowadays, none of the three groups of FRs (RFRs, AFRs, and CFRs) can be considered to be ideal additives for PUFs. The application of RFRs is limited by relatively low flame-retardant efficiency [40] and the need to elaborate the complicated synthetic routes, increasing the final product price [32,35]. AFR and CFR introduction in fire retardant PUFs is cost effective and does not require complicated production [38,40,43]. Nevertheless, to provide a satisfactory fire retardance level, the AFR mass fraction usually should be rather huge (up to 50%) which may affect the viscosity of PUF components, and hence, complicate filler implementation in industrial production [40]. Moreover, unlike RFRs, AFRs may suffer from poor interfacial compatibility with PU and poor dispersion, which could lead to filler migration during foam exploitation and deterioration of foam mechanical properties. The CFRs provide one of the highest levels of fire retardance; however, the durability of the protective coating as well as a possibility of industrial upscaling are questionable.

The application of nanomaterials can response to the challenges mentioned above. The application of AFR and CFR based on nanosized materials combined with other FRs leads to considerable enhancement of the final fire retardant effectivity (e.g., the combination of EG with a phosphorous-containing FR such as DOPO [136] or DPP [142]). Thus, for instance, by employing such synergy between different components of a multicomponent FR system, one can considerably decrease the mass fraction of AFR in composite PUF. FR encapsulation in various nanomaterials is one of the approaches to improve FR binning with PU (e.g., [146,183]). Chemical modification of nanosized additives is an alternative way to enhance their dispersion quality and compatibility with the polymer matrix, thus, improving mechanical properties of the foam and overcoming the AFR migration issue (e.g., [97,145]). The chemical modification/grafting of nanomaterials can also reinforce CFR adhesion to the PUF surface (e.g., [144,164]). In general, the introduction of nanomaterials in CFRs helps to decrease thermal conductivity and to improve thermal/mechanical stability of both the protective coating and the barrier char layer during foam burning [40,45,46,63]. Again, a synergetic effect is observed when nanosized particles are applied with various FRs, resulting in considerable improvement of char layer formation and smoke suppression (e.g., [91,174]).

As noted above, the synergy between various components of multicomponent FR systems has the outmost importance in the production of fireproof PUF composites. The investigation of new combinations of already known FRs with new types of additives should be the main efforts of future studies and industry. Taking into consideration the outstanding mechanical and electrical properties of some of the abovementioned reviewed nanomaterials, special attention should be given to the production of multifunctional/smart PUF composites. Thus, the application of smart fire-resistant coatings that provide, at the same time, the flame alarm option [101,104,105,106,107] to PUFs is a good example of elaboration next-generation foam composite materials. Environmental issues are last but not the least. In this regard, incorporation of natural nanosized fillers (e.g., nanoclays, nanocellulose, etc.) in combination with natural FRs (e.g., chitosan) in the polymer matrix can simultaneously decrease production price and improve PUFs recyclability.

## 7. Conclusions

Despite their high commercial importance, PUFs are highly flammable polymer materials. Incorporation of nano-sized FR additives into foam structure yields nanocomposite material with improved thermal and mechanical stability and fire resistance. The present work summarized the recent progress (last 5 years) in the elaboration of nanocomposite fireproof PUFs. The main groups of nano-sized FR additives such as carbon nanomaterials (including CNTs, graphene, and EG), nanoclays (including one-dimensional halloysite and sepiolite and two-dimensional kaolinite and montmorillonite), inorganic NPs, and some layered nanomaterials are comprehensively reviewed, as well as methods used to integrate them into the polymer matrix.

It is noteworthy that, despite some usual improvements in the mechanical properties and thermal stability of nanocomposite foams after incorporation of nano-sized additives, the latter are usually insufficient for effective fire resistance (with the exception of EG). Nevertheless, nanomaterials have become a priceless component of complex AFR or CFR systems, providing synergetic effect with conventional FRs. In most cases, the fire resistance of PUFs modified with conventional FRs is lower than the fire resistance of foams containing hybrid FRs. Occasionally, the outstanding physical properties of nano-fillers (e.g., electrical conductivity) facilitate the creation of smart fireproof foam materials, for instance, nanocomposite PUFs with “fire-alarm” function. The presence of a surface charge offers opportunities for a variety of modifications of nanomaterials, transforming them into multifunctional nanocomposites. Obviously, elaboration of next-generation smart porous polymer materials relies on the incorporation of such complex nano-scale additives.

## Figures and Tables

**Table 1:** Some FRs applied to fire-resistant PUF production.

Halogen-Containing FRs	Phosphorous-Containing FRs	Nitrogen-Containing FRs	Other FRs
Cycloaliphatic compounds (e.g., HET acid, TCPA, HCCPD, HBCDD, HBCD, TBPA) Esters/ethers (PBDEs, DECA, DBDPO) Derivatives of aromatic compounds (e.g., TBBPA, TBP, pentachlorophenol) Other organic compounds (e.g., halogen-containing polyols and isocyanates, aliphatic compounds, polymers, paraffins)	Elemental red phosphorous Inorganic phosphates (e.g., APP) Organic phosphates (including aryl phosphates (e.g., TPP), alkyl phosphates (e.g., TBP), bisphosphates (e.g., RDP, BDP)) Chlorinated phosphates (e.g., TCEP, TCPP, TDCP) Phosphinates (e.g., DEPAL) Phosphine oxides (e.g., DOPO)	Melamine and its derivatives (e.g., MCA, melamine homologs) Inorganic compounds (e.g., APP, ammonium biborate/pentaborate) Organic compounds (e.g., urea, EUF)	Metals hydroxides (e.g., ATH, MDH) Metals salts (e.g., zinc borate, zinc stannate, zinc hydroxystannate, zinc carbonate, calcium carbonate, manganese carbonate, ammonium carbonate, antimony trioxide, arsenic oxide, calcium sulfate) Expandable graphite Carbon nanomaterials Clays (e.g., montmorillonite, kaolinite)

**Table 4:** Results of the CCT and the LOI test of various NC-containing composite PUFs.

FR	FR Type	LOI (%)	Δ pHRR (%)	Δ THR (%)	Δ TSR (%)	Ref.
SA-sepiolite/PEI (6 BL)	CFR	–	−76	−27	–	[161]
BPEI-HNT/PAA-HNT (5 BL)	CFR	–	−62	+2	−60	[163]
PEI/APP/HNT	CFR	–	−53	−3	+200	[166]
Kaolinite	AFR	–	−25	−29	−65	[170]
VMT/CS (8 BL)	CFR	–	−53	−18	−63	[171]
Mica-PAA/CS (8 BL)	CFR	–	−54	–	−76	[172]
OMMT/APP-TPP	AFR	–	−34	−2	–	[180]
PAA/LDH/BPEI/Na-MMT	CFR	–	−31	−21	–	[177]
Cationic starch/MMT (5 BL)	CFR	–	−23	−58	–	[173]
Salvia filler/MMT	AFR	21	−6	−3	−11	[176]
MMT/CS/poly-D-lysine (5 TL)@CS-PA	CFR	–	−73	−2	+140	[174]
APP/modified-OMMT	AFR	–	−51	+6	−40	[181]
LDH/GO-SC4A (9 BL)	CFR	–	−29	−12	–	[178]
HT-FR-047	AFR	30	–	–	–	[179]
SuBC4A-LDH	AFR	23	−44	−29	–	[182]
LDH-H_2_PO_4_^−^	AFR	25	−68	−84	−49	[183]

**Table 5:** Results of the CCT and the LOI test of composite PUFs containing inorganic nanomaterials.

FR	FR Type	LOI (%)	Δ pHRR (%)	Δ THR (%)	Δ TSR (%)	Ref.
SiO_2_ NPs	CFR	20	−55	−21	−64	[187]
SiO_2_ NPs	CFR	33	−40	−29	–	[189]
Al_2_O_3_ NPs	CFR	–	−80	–	–	[194]
12EG/3Zr-AMP	AFR	31	−74	−62	–	[120]
DOPO@TiO_2_ NPs	AFR	–	−21	−7	–	[188]
DOPO@SiO_2_ NPs	AFR	–	−56	–	–	[190]
CuO NPs	AFR	22	–	–	–	[191]
ZnO NPs/ATTH	AFR	21	–	–	–	[192]
MgO NPS/ATH		21	–	–	–	
Fe_3_O_4_@ZIF-8	CFR	–	−69	–	–	[193]
MXene/CS (8BL)	CFR	–	−57	−66	−71	[195]

## Data Availability

Not applicable.

## References

[1] Ionescu M. (2005). Chemistry and Technology of Polyols for Polyurethanes.

[2] Market Volume of Polyurethane Worldwide from 2015 to 2025, with a Forecast for 2022 to 2029. https://www.statista.com/statistics/720341/global-polyurethane-market-size-forecast/#:~:text=The%20global%20market%20volume%20of,million%20metric%20tons%20in%202021.

[3] Palm E., Svensson Myrin E. (2018). Mapping the Plastics System and Its Sustainability Challenges.

[4] Polyurethane Production, Pricing and Market Demand. https://www.statista.com/statistics/720449/globalpolyurethane-market-size-forecast/.

[5] Oertel G. (1993). Polyurethane Handbook.

[6] Szycher M. (2006). Szycher’s Handbook of Polyurethanes.

[7] Ashida K. (2007). Polyurethane and Related Foams Chemistry and Technology.

[8] Lee L., Zeng C., Cao X., Han X., Shen J., Xu G. (2005). Polymer nanocomposite foams. Compos. Sci. Technol..

[9] Prisacariu C. (2011). Polyurethane Elastomers: From Morphology to Mechanical Aspects.

[10] Król P. (2008). Linear Polyurethanes: Synthesis Methods, Chemical Structures, Properties and Applications.

[11] Guide P. (2003). MDI and TDI: Safety, Health and the Environment. A Source Book and Practical Guide.

[12] Gama N., Ferreira A., Barros-Timmons A. (2018). Polyurethane Foams: Past, Present, and Future. Materials.

[13] Jiang L., Ren Z., Zhao W., Liu W., Liu H., Zhu C. (2018). Synthesis and structure/properties characterizations of four polyurethane model hard segments. R. Soc. Open Sci..

[14] Javni I., Zhang W., Petrović Z.S. (2003). Effect of different isocyanates on the properties of soy-based polyurethanes. J. Appl. Polym. Sci..

[15] Maiuolo L., Olivito F., Ponte F., Algieri V., Tallarida M.A., Tursi A., Chidichimo G., Sicilia E., De Nino A. (2021). A novel catalytic two-step process for the preparation of rigid polyurethane foams: Synthesis, mechanism and computational studies. React. Chem. Eng..

[16] Ates M., Karadag S., Eker A.A., Eker B. (2022). Polyurethane foam materials and their industrial applications. Polym. Int..

[17] Vollrath A., Hohl C., Seiler H.G. (1995). Trace analysis of chlorofluorocarbons/partially halogenated chlorofluorohydrocarbons (CFC/HCFC) in polymeric foams by headspace capillary gas chromatography with electron-capture detection or ion trap detection combined with preconcentration on a cold trap. Fresenius J. Anal. Chem..

[18] Petrović Z.S., Ferguson J. (1991). Polyurethane elastomers. Prog. Polym. Sci..

[19] Kausar A. (2018). Polyurethane Composite Foams in High-Performance Applications: A Review. Polym. Plast. Technol. Eng..

[20] Heintz A.M., Duffy D.J., Hsu S.L., Suen W., Chu W., Paul C.W. (2003). Effects of Reaction Temperature on the Formation of Polyurethane Prepolymer Structures. Macromolecules.

[21] Sattar R., Kausar A., Siddiq M. (2015). Thermal, mechanical and electrical studies of novel shape memory polyurethane/polyaniline blends. Chin. J. Polym. Sci..

[22] Yang C., Fischer L., Maranda S., Worlitschek J. (2015). Rigid polyurethane foams incorporated with phase change materials: A state-of-the-art review and future research pathways. Energy Build..

[23] Mondal P., Khakhar D.V. (2004). Regulation of Cell Structure in Water Blown Rigid Polyurethane Foam. Macromol. Symp..

[24] Ge C., Lian D., Cui S., Gao J., Lu J. (2019). Highly Selective CO2 Capture on Waste Polyurethane Foam-Based Activated Carbon. Processes.

[25] Dacewicz E., Grzybowska-Pietras J. (2021). Polyurethane Foams for Domestic Sewage Treatment. Materials.

[26] Van Minnen B., van Leeuwen M.B.M., Stegenga B., Zuidema J., Hissink C.E., van Kooten T.G., Bos R.R.M. (2005). Short-term in vitro and in vivo biocompatibility of a biodegradable polyurethane foam based on 1,4-butanediisocyanate. J. Mater. Sci. Mater. Med..

[27] Sakurai A., Hashikawa K., Yokoo S., Terashi H., Tahara S. (2007). Simple Dressing Technique Using Polyurethane Foam for Fixation of Skin Grafts. Dermatol. Surg..

[28] Baer G., Wilson T.S., Matthews D.L., Maitland D.J. (2007). Shape-memory behavior of thermally stimulated polyurethane for medical applications. J. Appl. Polym. Sci..

[29] Das A., Mahanwar P. (2020). A brief discussion on advances in polyurethane applications. Adv. Ind. Eng. Polym. Res..

[30] Grzęda D., Węgrzyk G., Leszczyńska M., Szczepkowski L., Gloc M., Ryszkowska J. (2021). Viscoelastic Polyurethane Foams for Use as Auxiliary Materials in Orthopedics. Materials.

[31] Visakh P.M., Semkin A.O., Rezaev I.A., Fateev A.V. (2019). Review on soft polyurethane flame retardant. Constr. Build. Mater..

[32] Chattopadhyay D.K., Webster D.C. (2009). Thermal stability and flame retardancy of polyurethanes. Prog. Polym. Sci..

[33] Levchik S.V., Weil E.D. (2004). Thermal decomposition, combustion and fire-retardancy of polyurethanes—A review of the recent literature. Polym. Int..

[34] Shen J., Liang J., Lin X., Lin H., Yu J., Wang S. (2021). The Flame-Retardant Mechanisms and Preparation of Polymer Composites and Their Potential Application in Construction Engineering. Polymers.

[35] Sing H., Jain A.K. (2008). Ignition, combustion, toxicity, and fire retardancy of polyurethane foams: A comprehensive review. J. Appl. Polym. Sci..

[36] Liu X., Hao J., Gaan S. (2016). Recent studies on the decomposition and strategies of smoke and toxicity suppression for polyurethane based materials. RSC Adv..

[37] Schrock A.K., Solis R., Beal G.E., Skorpenske R.G., Parrish D.P. (1990). The Influence of Polymer Morphology on the Combustion of Melamine Filled Flexible Foams. J. Fire Sci..

[38] Wang J.Q., Chow W.K. (2005). A brief review on fire retardants for polymeric foams. J. Appl. Polym. Sci..

[39] Lefebvre J., le Bras M., Bastin B., Paleja R., Delobel R. (2003). Flexible Polyurethane Foams: Flammability. J. Fire Sci..

[40] Zhu M., Ma Z., Liu L., Zhang J., Huo S., Song P. (2022). Recent advances in fire-retardant rigid polyurethane foam. J. Mater. Sci. Technol..

[41] Yang H., Yu B., Song P., Maluk C., Wang H. (2019). Surface-coating engineering for flame retardant flexible polyurethane foams: A critical review. Compos. Part B Eng..

[42] Weil E.D., Levchik S.V. (2004). Commercial Flame Retardancy of Polyurethanes. J. Fire Sci..

[43] Ali M.H.M., Rahman H.A., Amirnordin S.H., Khan N.A. (2018). Eco-Friendly Flame-Retardant Additives for Polyurethane Foams: A Short Review. KEM.

[44] Raji A.M., Hambali H.U., Khan Z.I., Mohamad Z.B., Azman H., Ogabi R. (2023). Emerging trends in flame retardancy of rigid polyurethane foam and its composites: A review. J. Cell. Plast..

[45] Jimenez M., Duquesne S., Bourbigot S. (2006). Intumescent fire protective coating: Toward a better understanding of their mechanism of action. Thermochim. Acta.

[46] Yadav A., de Souza F.M., Dawsey T., Gupta R.K. (2022). Recent Advancements in Flame-Retardant Polyurethane Foams: A Review. Ind. Eng. Chem. Res..

[47] Laoutid F., Bonnaud L., Alexandre M., Lopez-Cuesta J.-M., Dubois P. (2009). New prospects in flame retardant polymer materials: From fundamentals to nanocomposites. Mater. Sci. Eng. R Rep..

[48] Dasari A., Yu Z.-Z., Cai G.-P., Mai Y.-W. (2013). Recent developments in the fire retardancy of polymeric materials. Prog. Polym. Sci..

[49] Hull T.R., Witkowski A., Hollingbery L. (2011). Fire retardant action of mineral fillers. Polym. Degrad. Stab..

[50] Modesti M., Lorenzetti A., Simioni F., Camino G. (2002). Expandable graphite as an intumescent flame retardant in polyisocyanurate–polyurethane foams. Polym. Degrad. Stab..

[51] Dong F., Wang Y., Wang S., Shaghaleh H., Sun P., Huang X., Xu X., Wang S., Liu H. (2021). Flame-retarded polyurethane foam conferred by a bio-based nitrogen-phosphorus-containing flame retardant. React. Funct. Polym..

[52] Jasinski E., Bounor-Legaré V., Taguet A., Beyou E. (2021). Influence of halloysite nanotubes onto the fire properties of polymer based composites: A review. Polym. Degrad. Stab..

[53] Kuranchie C., Yaya A., Bensah Y.D. (2021). The effect of natural fibre reinforcement on polyurethane composite foams—A review. Sci. Afr..

[54] Fu S.-Y., Sun Z., Huang P., Li Y.-Q., Hu N. (2019). Some basic aspects of polymer nanocomposites: A critical review. Nano Mater. Sci..

[55] Fawaz J., Mittal V., Mittal V. (2014). Synthesis of Polymer Nanocomposites: Review of Various Techniques. Synthesis Techniques for Polymer Nanocomposites.

[56] Yang F., Xie M., Yudi Z., Xu X. (2021). Effect of multi-walled carbon nanotubes with different diameters on morphology and thermal and mechanical properties of flexible polyurethane foams. Cell. Polym..

[57] Pan H., Pan Y., Wang W., Song L., Hu Y., Liew K.M. (2014). Synergistic Effect of Layer-by-Layer Assembled Thin Films Based on Clay and Carbon Nanotubes to Reduce the Flammability of Flexible Polyurethane Foam. Ind. Eng. Chem. Res..

[58] Levchik S.V., Morgan A.B., Wilkie C.A. (2007). Introduction to Flame Retardancy and Polymer Flammability. Flame Retardant Polymer Nanocomposites.

[59] Hejna A. (2021). Clays as Inhibitors of Polyurethane Foams’ Flammability. Materials.

[60] Kashiwagi T., Du F., Douglas J.F., Winey K.I., Harris R.H., Shields J.R. (2005). Nanoparticle networks reduce the flammability of polymer nanocomposites. Nat. Mater..

[61] Yang Y., Palencia J.L.D., Wang N., Jiang Y., Wang D.-Y. (2021). Nanocarbon-Based Flame Retardant Polymer Nanocomposites. Molecules.

[62] Qiu X., Li Z., Li X., Zhang Z. (2018). Flame retardant coatings prepared using layer by layer assembly: A review. Chem. Eng. J..

[63] Liu Q., Gao S., Zhao Y., Tao W., Yu X., Zhi M. (2021). Review of layer-by-layer self-assembly technology for fire protection of flexible polyurethane foam. J. Mater. Sci..

[64] Navidfar A., Sancak A., Yildirim K.B., Trabzon L. (2018). A Study on Polyurethane Hybrid Nanocomposite Foams Reinforced with Multiwalled Carbon Nanotubes and Silica Nanoparticles. Polym. Plast. Technol. Eng..

[65] Yaghoubi A., Nikje M.M.A. (2018). Silanization of multi-walled carbon nanotubes and the study of its effects on the properties of polyurethane rigid foam nanocomposites. Compos. Part A Appl. Sci. Manuf..

[66] Wang X., Li H., Wang T., Niu X., Wang Y., Xu S., Jiang Y., Chen L., Liu H. (2022). Flexible and high-performance piezoresistive strain sensors based on multi-walled carbon nanotubes@polyurethane foam. RSC Adv..

[67] Holder K.M., Cain A.A., Plummer M.G., Stevens B.E., Odenborg P.K., Morgan A.B., Grunlan J.C. (2016). Carbon Nanotube Multilayer Nanocoatings Prevent Flame Spread on Flexible Polyurethane Foam. Macromol. Mater. Eng..

[68] Kim J., Jang J., Yun S., Kim H.D., Byun Y.Y., Park Y.T., Song J.I., Cho C. (2021). Synergistic Flame Retardant Effects of Carbon Nanotube-Based Multilayer Nanocoatings. Macromol. Mater. Eng..

[69] Kim Y.S., Davis R. (2014). Multi-walled carbon nanotube layer-by-layer coatings with a trilayer structure to reduce foam flammability. Thin Solid Films.

[70] Shimazaki Y., Mitsuishi M., Ito S., Yamamoto M. (1997). Preparation of the Layer-by-Layer Deposited Ultrathin Film Based on the Charge-Transfer Interaction. Langmuir.

[71] Benten H., Ogawa M., Ohkita H., Ito S. (2008). Design of Multilayered Nanostructures and Donor-Acceptor Interfaces in Solution-Processed Thin-Film Organic Solar Cells: Design of Multilayered Nanostructures for Solar Cells. Adv. Funct. Mater..

[72] Stockton W.B., Rubner M.F. (1997). Molecular-Level Processing of Conjugated Polymers. 4. Layer-by-Layer Manipulation of Polyaniline via Hydrogen-Bonding Interactions. Macromolecules.

[73] Wang L., Cui S., Wang Z., Zhang X., Jiang M., Chi L., Fuchs H. (2000). Multilayer Assemblies of Copolymer PSOH and PVP on the Basis of Hydrogen Bonding. Langmuir.

[74] Fang M., Kaschak D.M., Sutorik A.C., Mallouk T.E. (1997). A “Mix and Match” Ionic−Covalent Strategy for Self-Assembly of Inorganic Multilayer Films. J. Am. Chem. Soc..

[75] Ichinose I., Kawakami T., Kunitake T. (1998). Alternate Molecular Layers of Metal Oxides and Hydroxyl Polymers Prepared by the Surface Sol-Gel Process. Adv. Mater..

[76] Cheng J., Niu S., Kang M., Liu Y., Zhang F., Qu W., Guan Y., Li S. (2022). The thermal behavior and flame retardant performance of phase change material microcapsules with modified carbon nanotubes. Energy.

[77] Zammarano M., Krämer R.H., Harris R., Ohlemiller T.J., Shields J.R., Rahatekar S.S., Lacerda S., Gilman J.W. (2008). Flammability reduction of flexible polyurethane foams via carbon nanofiber network formation. Polym. Adv. Technol..

[78] Kim Y.S., Davis R., Cain A.A., Grunlan J.C. (2011). Development of layer-by-layer assembled carbon nanofiber-filled coatings to reduce polyurethane foam flammability. Polymer.

[79] Geim A.K., Novoselov K.S. (2007). The rise of graphene. Nat. Mater..

[80] Ababsa H.S., Safidine Z., Mekki A., Grohens Y., Ouadah A., Chabane H. (2021). Fire behavior of flame-retardant polyurethane semi-rigid foam in presence of nickel (II) oxide and graphene nanoplatelets additives. J. Polym. Res..

[81] Jamsaz A., Goharshadi E.K. (2022). Graphene-based flame-retardant polyurethane: A critical review. Polym. Bull..

[82] Pan H., Yu B., Wang W., Pan Y., Song L., Hu Y. (2016). Comparative study of layer by layer assembled multilayer films based on graphene oxide and reduced graphene oxide on flexible polyurethane foam: Flame retardant and smoke suppression properties. RSC Adv..

[83] Pan H., Lu Y., Song L., Zhang X., Hu Y. (2016). Construction of layer-by-layer coating based on graphene oxide/β-FeOOH nanorods and its synergistic effect on improving flame retardancy of flexible polyurethane foam. Compos. Sci. Technol..

[84] Zhang X., Shen Q., Zhang X., Pan H., Lu Y. (2016). Graphene oxide-filled multilayer coating to improve flame-retardant and smoke suppression properties of flexible polyurethane foam. J. Mater. Sci..

[85] Kim H., Kim D.W., Vasagar V., Ha H., Nazarenko S., Ellison C.J. (2018). Polydopamine-Graphene Oxide Flame Retardant Nanocoatings Applied via an Aqueous Liquid Crystalline Scaffold. Adv. Funct. Mater..

[86] Carosio F., Maddalena L., Gomez J., Saracco G., Fina A. (2018). Graphene Oxide Exoskeleton to Produce Self-Extinguishing, Nonignitable, and Flame Resistant Flexible Foams: A Mechanically Tough Alternative to Inorganic Aerogels. Adv. Mater. Interfaces.

[87] Maddalena L., Carosio F., Gomez J., Saracco G., Fina A. (2018). Layer-by-layer assembly of efficient flame retardant coatings based on high aspect ratio graphene oxide and chitosan capable of preventing ignition of PU foam. Polym. Degrad. Stab..

[88] Li Y., Cao C.-F., Li S.-N., Huang N.-J., Mao M., Zhang J.-W., Wang P.-H., Guo K.-Y., Gong L.-X., Zhang G.-D. (2019). In situ reactive self-assembly of a graphene oxide nano-coating in polymer foam materials with synergistic fire shielding properties. J. Mater. Chem. A.

[89] Jamsaz A., Goharshadi E.K. (2020). Flame retardant, superhydrophobic, and superoleophilic reduced graphene oxide/orthoaminophenol polyurethane sponge for efficient oil/water separation. J. Mol. Liq..

[90] Yuen A.C.Y., Chen T.B.Y., Wang C., Wei W., Kabir I., Vargas J.B., Chan Q.N., Kook S., Yeoh G.H. (2020). Utilising genetic algorithm to optimise pyrolysis kinetics for fire modelling and characterisation of chitosan/graphene oxide polyurethane composites. Compos. Part B Eng..

[91] Maddalena L., Gomez J., Fina A., Carosio F. (2021). Effects of Graphite Oxide Nanoparticle Size on the Functional Properties of Layer-by-Layer Coated Flexible Foams. Nanomaterials.

[92] Wu Q., Zhang J., Wang S., Chen B., Feng Y., Pei Y., Yan Y., Tang L., Qiu H., Wu L. (2021). Exceptionally flame-retardant flexible polyurethane foam composites: Synergistic effect of the silicone resin/graphene oxide coating. Front. Chem. Sci. Eng..

[93] Meng D., Liu X., Wang S., Sun J., Li H., Wang Z., Gu X., Zhang S. (2021). Self-healing polyelectrolyte complex coating for flame retardant flexible polyurethane foam with enhanced mechanical property. Compos. Part B Eng..

[94] Qiu X., Kundu C.K., Li Z., Li X., Zhang Z. (2019). Layer-by-layer-assembled flame-retardant coatings from polydopamine-induced in situ functionalized and reduced graphene oxide. J. Mater. Sci..

[95] Pan H., Lu Y., Song L., Zhang X., Hu Y. (2016). Fabrication of binary hybrid-filled layer-by-layer coatings on flexible polyurethane foams and studies on their flame-retardant and thermal properties. RSC Adv..

[96] Gao M., Li J., Zhou X. (2019). A flame retardant rigid polyurethane foam system including functionalized graphene oxide. Polym. Compos..

[97] Cao Z.-J., Liao W., Wang S.-X., Zhao H.-B., Wang Y.-Z. (2019). Polyurethane foams with functionalized graphene towards high fire-resistance, low smoke release, superior thermal insulation. Chem. Eng. J..

[98] Chen X., Li J., Gao M. (2019). Thermal Degradation and Flame Retardant Mechanism of the Rigid Polyurethane Foam Including Functionalized Graphene Oxide. Polymers.

[99] Sałasińska K., Leszczyńska M., Celiński M., Kozikowski P., Kowiorski K., Lipińska L. (2021). Burning Behaviour of Rigid Polyurethane Foams with Histidine and Modified Graphene Oxide. Materials.

[100] Gao M., Wang T., Chen X., Zhang X., Yi D., Qian L., You R. (2022). Preparation of ionic liquid multifunctional graphene oxide and its effect on decrease fire hazards of flexible polyurethane foam. J. Therm. Anal. Calorim..

[101] Wu Q., Gong L.-X., Li Y., Cao C.-F., Tang L.-C., Wu L., Zhao L., Zhang G.-D., Li S.-N., Gao J. (2018). Efficient Flame Detection and Early Warning Sensors on Combustible Materials Using Hierarchical Graphene Oxide/Silicone Coatings. ACS Nano.

[102] Guo K.-Y., Wu Q., Mao M., Chen H., Zhang G.-D., Zhao L., Gao J.-F., Song P., Tang L.-C. (2020). Water-based hybrid coatings toward mechanically flexible, super-hydrophobic and flame-retardant polyurethane foam nanocomposites with high-efficiency and reliable fire alarm response. Compos. Part B Eng..

[103] Wu Q., Liu C., Tang L., Yan Y., Qiu H., Pei Y., Sailor M.J., Wu L. (2021). Stable electrically conductive, highly flame-retardant foam composites generated from reduced graphene oxide and silicone resin coatings. Soft Matter..

[104] Yu Z.-R., Mao M., Li S.-N., Xia Q.-Q., Cao C.-F., Zhao L., Zhang G.-D., Zheng Z.-J., Gao J.-F., Tang L.-C. (2021). Facile and green synthesis of mechanically flexible and flame-retardant clay/graphene oxide nanoribbon interconnected networks for fire safety and prevention. Chem. Eng. J..

[105] Cao C.-F., Yu B., Chen Z.-Y., Qu Y.-X., Li Y.-T., Shi Y.-Q., Ma Z.-W., Sun F.-N., Pan Q.-H., Tang L.-C. (2022). Fire Intumescent, High-Temperature Resistant, Mechanically Flexible Graphene Oxide Network for Exceptional Fire Shielding and Ultra-Fast Fire Warning. Nano-Micro Lett..

[106] Chen Z., Chen W., Liu P., Liu Y., Liu Z. (2021). A multifunctional polyurethane sponge based on functionalized graphene oxide and carbon nanotubes for highly sensitive and super durable fire alarming. Compos. Part A Appl. Sci. Manuf..

[107] Ma Z., Zhang J., Liu L., Zheng H., Dai J., Tang L.-C., Song P. (2022). A highly fire-retardant rigid polyurethane foam capable of fire-warning. Compos. Commun..

[108] Cai M., Thorpe D., Adamson D.H., Schniepp H.C. (2012). Methods of graphite exfoliation. J. Mater. Chem..

[109] Papaspyrides C.D., Kiliaris P. (2014). Polymer Green Flame Retardants.

[110] Chao C., Gao M., Chen S. (2018). Expanded graphite: Borax synergism in the flame-retardant flexible polyurethane foams. J. Anal. Calorim..

[111] Gama N.V., Silva R., Mohseni F., Davarpanah A., Amaral V.S., Ferreira A., Barros-Timmons A. (2018). Enhancement of physical and reaction to fire properties of crude glycerol polyurethane foams filled with expanded graphite. Polym. Test..

[112] Li J., Mo X., Li Y., Zou H., Liang M., Chen Y. (2018). Influence of expandable graphite particle size on the synergy flame retardant property between expandable graphite and ammonium polyphosphate in semi-rigid polyurethane foam. Polym. Bull..

[113] Li L., Chen Y., Qian L., Xu B., Xi W. (2018). Addition flame-retardant effect of nonreactive phosphonate and expandable graphite in rigid polyurethane foams. J. Appl. Polym. Sci..

[114] Liu D.-Y., Zhao B., Wang J.-S., Liu P.-W., Liu Y.-Q. (2018). Flame retardation and thermal stability of novel phosphoramide/expandable graphite in rigid polyurethane foam: Research Article. J. Appl. Polym. Sci..

[115] Rao W.-H., Liao W., Wang H., Zhao H.-B., Wang Y.-Z. (2018). Flame-retardant and smoke-suppressant flexible polyurethane foams based on reactive phosphorus-containing polyol and expandable graphite. J. Hazard. Mater..

[116] Wang S., Qian L., Xin F. (2018). The synergistic flame-retardant behaviors of pentaerythritol phosphate and expandable graphite in rigid polyurethane foams. Polym. Compos..

[117] Acuña P., Li Z., Santiago-Calvo M., Villafañe F., Rodríguez-Perez M., Wang D.-Y. (2019). Influence of the Characteristics of Expandable Graphite on the Morphology, Thermal Properties, Fire Behaviour and Compression Performance of a Rigid Polyurethane Foam. Polymers.

[118] Acuña P., Santiago-Calvo M., Villafañe F., Rodríguez-Perez M.A., Rosas J., Wang D. (2019). Impact of expandable graphite on flame retardancy and mechanical properties of rigid polyurethane foam. Polym. Compos..

[119] Chen Y., Luo Y., Guo X., Chen L., Xu T., Jia D. (2019). Structure and Flame-Retardant Actions of Rigid Polyurethane Foams with Expandable Graphite. Polymers.

[120] Liu L., Wang Z., Zhu M. (2019). Flame retardant, mechanical and thermal insulating properties of rigid polyurethane foam modified by nano zirconium amino-tris-(methylenephosphonate) and expandable graphite. Polym. Degrad. Stab..

[121] Qian L., Li L., Chen Y., Xu B., Qiu Y. (2019). Quickly self-extinguishing flame retardant behavior of rigid polyurethane foams linked with phosphaphenanthrene groups. Compos. Part B Eng..

[122] Thi N.H., Pham D.L., Hanh N.T., Oanh H.T., Duong T.H.Y., Nguyen T.N., Tuyen N.D., Phan D.L., Trinh H.T., Nguyen H.T. (2019). Influence of Organoclay on the Flame Retardancy and Thermal Insulation Property of Expandable Graphite/Polyurethane Foam. J. Chem..

[123] Xi W., Qian L., Li L. (2019). Flame Retardant Behavior of Ternary Synergistic Systems in Rigid Polyurethane Foams. Polymers.

[124] Yao W., Zhang D., Zhang Y., Fu T., Guan D., Dou Y. (2019). Synergistic Flame Retardant Effects of Expandable Graphite and Ammonium Polyphosphate in Water-Blow Polyurethane Foam. Adv. Mater. Sci. Eng..

[125] Acuña P., Lin X., Calvo M.S., Shao Z., Pérez N., Villafañe F., Rodríguez-Pérez M.Á., Wang D.-Y. (2020). Synergistic effect of expandable graphite and phenylphosphonic-aniline salt on flame retardancy of rigid polyurethane foam. Polym. Degrad. Stab..

[126] Akdogan E., Erdem M., Ureyen M.E., Kaya M. (2020). Synergistic effects of expandable graphite and ammonium pentaborate octahydrate on the flame-retardant, thermal insulation, and mechanical properties of rigid polyurethane foam. Polym. Compos..

[127] Liu C., Zhang P., Shi Y., Rao X., Cai S., Fu L., Feng Y., Wang L., Zheng X., Yang W. (2020). Enhanced Fire Safety of Rigid Polyurethane Foam via Synergistic Effect of Phosphorus/Nitrogen Compounds and Expandable Graphite. Molecules.

[128] Strąkowska A., Członka S., Konca P., Strzelec K. (2020). New Flame Retardant Systems Based on Expanded Graphite for Rigid Polyurethane Foams. Appl. Sci..

[129] Yun G.W., Lee J.H., Kim S.H. (2020). Flame retardant and mechanical properties of expandable graphite/polyurethane foam composites containing iron phosphonate dopamine-coated cellulose. Polym. Compos..

[130] Zhang W., Lei Y., Li X., Shao H., Xu W., Li D. (2020). A facile, environmentally and friendly flame-retardant: Synergistic flame retardant property of polyurethane rigid foam. Mater. Lett..

[131] Zhang Z., Li D., Xu M., Li B. (2020). Synthesis of a novel phosphorus and nitrogen-containing flame retardant and its application in rigid polyurethane foam with expandable graphite. Polym. Degrad. Stab..

[132] Chan Y.Y., Ma C., Zhou F., Hu Y., Schartel B. (2021). Flame retardant flexible polyurethane foams based on phosphorous soybean-oil polyol and expandable graphite. Polym. Degrad. Stab..

[133] Hu Y., Zhou Z., Li S., Yang D., Zhang S., Hou Y. (2021). Flame Retarded Rigid Polyurethane Foams Composites Modified by Aluminum Diethylphosphinate and Expanded Graphite. Front. Mater..

[134] Wang J., Xu B., Wang X., Liu Y. (2021). A phosphorous-based bi-functional flame retardant for rigid polyurethane foam. Polym. Degrad. Stab..

[135] Xu J., Wu Y., Zhang B., Zhang G. (2021). Synthesis and synergistic flame-retardant effects of rigid polyurethane foams used reactive DOPO -based polyols combination with expandable graphite. J. Appl. Polym. Sci..

[136] Chan Y.Y., Ma C., Zhou F., Hu Y., Schartel B. (2022). A liquid phosphorous flame retardant combined with expandable graphite or melamine in flexible polyurethane foam. Polym. Adv. Technol..

[137] Chan Y.Y., Schartel B. (2022). It Takes Two to Tango: Synergistic Expandable Graphite–Phosphorus Flame Retardant Combinations in Polyurethane Foams. Polymers.

[138] Liu M., Feng Z., Zhao R., Wang B., Deng D., Zhou Z., Yang Y., Liu X., Liu X., Tang G. (2022). Enhancement of fire performance for rigid polyurethane foam composites by incorporation of aluminum hypophosphite and expanded graphite. Polym. Bull..

[139] Wang X., Sun Y., Sheng J., Geng T., Turng L., Guo Y., Liu X., Liu C. (2022). Effects of expandable graphite on the flame-retardant and mechanical performances of rigid polyurethane foams. J. Phys. Condens. Matter..

[140] Yang R., Gu G., Li M., Li J. (2022). Preparation of flame-retardant rigid polyurethane foam with bio-based phosphorus-containing polyols and expandable graphite. J Appl. Polym. Sci..

[141] Yang Y., Sun P., Sun J., Wen P., Zhang S., Kan Y., Liu X., Tang G. (2022). Enhanced flame retardancy of rigid polyurethane foam via iron tailings and expandable graphite. J. Mater. Sci..

[142] Wang H., Liu Q., Li H., Zhang H., Yan S. (2023). Flame-Retardant and Smoke-Suppressant Flexible Polyurethane Foams Based on Phosphorus-Containing Polyester Diols and Expandable Graphite. Polymers.

[143] Wang S., Wang X., Wang X., Li H., Sun J., Sun W., Yao Y., Gu X., Zhang S. (2020). Surface coated rigid polyurethane foam with durable flame retardancy and improved mechanical property. Chem. Eng. J..

[144] Wong E.H.H., Fan K.W., Lei L., Wang C., Baena J.C., Okoye H., Fam W., Zhou D., Oliver S., Khalid A. (2021). Fire-Resistant Flexible Polyurethane Foams via Nature-Inspired Chitosan-Expandable Graphite Coatings. ACS Appl. Polym. Mater..

[145] Zhang W., Zhao Z., Lei Y. (2021). Flame retardant and smoke-suppressant rigid polyurethane foam based on sodium alginate and aluminum diethylphosphite. Des. Monomers Polym..

[146] Wang Y., Wang F., Dong Q., Yuan W., Liu P., Ding Y., Zhang S., Yang M., Zheng G. (2018). Expandable graphite encapsulated by magnesium hydroxide nanosheets as an intumescent flame retardant for rigid polyurethane foams. J. Appl. Polym. Sci..

[147] Wang Y., Wang F., Dong Q., Xie M., Liu P., Ding Y., Zhang S., Yang M., Zheng G. (2017). Core-shell expandable graphite @ aluminum hydroxide as a flame-retardant for rigid polyurethane foams. Polym. Degrad. Stab..

[148] Pang X., Xin Y., Shi X., Xu J. (2019). Effect of different size-modified expandable graphite and ammonium polyphosphate on the flame retardancy, thermal stability, physical, and mechanical properties of rigid polyurethane foam. Polym. Eng. Sci..

[149] Yang Y., Dai Z., Liu M., Jiang H., Fan C., Wang B., Tang G., Wang H. (2021). Flame retardant rigid polyurethane foam composites based on microencapsulated ammonium polyphosphate and microencapsulated expanded graphite. J. Macromol. Sci. Part A.

[150] Cheng J., Qu W., Sun S. (2019). Mechanical properties improvement and fire hazard reduction of expandable graphite microencapsulated in rigid polyurethane foams. Polym. Compos..

[151] Chen Y., Luo Y., Guo X., Chen L., Jia D. (2020). The Synergistic Effect of Ionic Liquid-Modified Expandable Graphite and Intumescent Flame-Retardant on Flame-Retardant Rigid Polyurethane Foams. Materials.

[152] Xiong W., Liu H., Tian H., Wu J., Xiang A., Wang C., Ma S., Wu Q. (2020). Mechanical and flame-resistance properties of polyurethane-imide foams with different-sized expandable graphite. Polym. Eng. Sci..

[153] Albdiry M., Yousif B., Ku H., Lau K. (2013). A critical review on the manufacturing processes in relation to the properties of nanoclay/polymer composites. J. Compos. Mater..

[154] Guo F., Aryana S., Han Y., Jiao Y. (2018). A Review of the Synthesis and Applications of Polymer–Nanoclay Composites. Appl. Sci..

[155] Rafiee R., Shahzadi R. (2019). Mechanical Properties of Nanoclay and Nanoclay Reinforced Polymers: A Review. Polym. Compos..

[156] Rajeshkumar G., Seshadri S.A., Ramakrishnan S., Sanjay M.R., Siengchin S., Nagaraja K.C. (2021). A comprehensive review on natural fiber/nano-clay reinforced hybrid polymeric composites: Materials and technologies. Polym. Compos..

[157] Alves L.R.P.S.T., Alves M.D.T.C., Honorio L.M.C., Moraes A.I., Silva-Filho E.C., Peña-Garcia R., Furtini M.B., da Silva D.A., Osajima J.A. (2022). Polyurethane/Vermiculite Foam Composite as Sustainable Material for Vertical Flame Retardant. Polymers.

[158] Cherednichenko K., Kopitsyn D., Batasheva S., Fakhrullin R. (2021). Probing Antimicrobial Halloysite/Biopolymer Composites with Electron Microscopy: Advantages and Limitations. Polymers.

[159] Khan Z.I., Habib U., Mohamad Z.B., Bin Rahmat A.R., Abdullah N.A.S.B. (2022). Mechanical and thermal properties of sepiolite strengthened thermoplastic polymer nanocomposites: A comprehensive review. Alex. Eng. J..

[160] Tian G., Han G., Wang F., Liang J. (2019). Sepiolite Nanomaterials: Structure, Properties and Functional Applications. Nanomaterials from Clay Minerals.

[161] Pan Y., Liu L., Cai W., Hu Y., Jiang S., Zhao H. (2019). Effect of layer-by-layer self-assembled sepiolite-based nanocoating on flame retardant and smoke suppressant properties of flexible polyurethane foam. Appl. Clay Sci..

[162] Goda E.S., Yoon K.R., El-sayed S.H., Hong S.E. (2018). Halloysite nanotubes as smart flame retardant and economic reinforcing materials: A review. Thermochim. Acta.

[163] Smith R.J., Holder K.M., Ruiz S., Hahn W., Song Y., Lvov Y.M., Grunlan J.C. (2018). Environmentally Benign Halloysite Nanotube Multilayer Assembly Significantly Reduces Polyurethane Flammability. Adv. Funct. Mater..

[164] Wu F., Pickett K., Panchal A., Liu M., Lvov Y. (2019). Superhydrophobic Polyurethane Foam Coated with Polysiloxane-Modified Clay Nanotubes for Efficient and Recyclable Oil Absorption. ACS Appl. Mater. Interfaces.

[165] Wu F., Zheng J., Ou X., Liu M. (2019). Two in One: Modified Polyurethane Foams by Dip-Coating of Halloysite Nanotubes with Acceptable Flame Retardancy and Absorbency. Macromol. Mater. Eng..

[166] Palen B., Kolibaba T.J., Brehm J.T., Shen R., Quan Y., Wang Q., Grunlan J.C. (2021). Clay-Filled Polyelectrolyte Complex Nanocoating for Flame-Retardant Polyurethane Foam. ACS Omega.

[167] Uddin F. (2008). Clays, Nanoclays, and Montmorillonite Minerals. Met. Mater. Trans. A.

[168] Neto J.C.D.M., Nascimento N.R.D., Bello R.H., de Verçosa L.A., Neto J.E., da Costa J.C.M., Diaz F.R.V. (2021). Kaolinite Review: Intercalation and Production of Polymer Nanocomposites. Eng. Sci..

[169] Conterosito E., Gianotti V., Palin L., Boccaleri E., Viterbo D., Milanesio M. (2018). Facile preparation methods of hydrotalcite layered materials and their structural characterization by combined techniques. Inorganica Chim. Acta.

[170] Agrawal A., Kaur R., Walia R.S. (2019). Investigation on flammability of rigid polyurethane foam-mineral fillers composite. Fire Mater..

[171] Lazar S., Carosio F., Davesne A.-L., Jimenez M., Bourbigot S., Grunlan J. (2018). Extreme Heat Shielding of Clay/Chitosan Nanobrick Wall on Flexible Foam. ACS Appl. Mater. Interfaces.

[172] Fahami A., Lee J., Lazar S., Grunlan J.C. (2020). Mica-Based Multilayer Nanocoating as a Highly Effective Flame Retardant and Smoke Suppressant. ACS Appl. Mater. Interfaces.

[173] Choi K.-W., Kim J.-W., Kwon T.-S., Kang S.-W., Song J.-I., Park Y.-T. (2021). Mechanically Sustainable Starch-Based Flame-Retardant Coatings on Polyurethane Foams. Polymers.

[174] Weldemhret T.G., Menge H.G., Lee D.-W., Park H., Lee J., Song J.I., Park Y.T. (2021). Facile deposition of environmentally benign organic-inorganic flame retardant coatings to protect flammable foam. Prog. Org. Coat..

[175] Akar A., Kızılcan N., Yivlik Y., Önen D. (2021). Alendronic acid bearing ketone-formaldehyde resin and clay nanocomposites for fire-retardant polyurethanes. J. Appl. Polym. Sci..

[176] Członka S., Kairytė A., Miedzińska K., Strąkowska A., Adamus-Włodarczyk A. (2021). Mechanically Strong Polyurethane Composites Reinforced with Montmorillonite-Modified Sage Filler (*Salvia officinalis* L.). Int. J. Mol. Sci..

[177] Yang Y.-H., Li Y.-C., Shields J., Davis R.D. (2015). Layer double hydroxide and sodium montmorillonite multilayer coatings for the flammability reduction of flexible polyurethane foams. J. Appl. Polym. Sci..

[178] Abrishamkar S., Mohammadi A., De La Vega J., Wang D.-Y., Kalali E.N. (2023). Layer-by-layer assembly of calixarene modified GO and LDH nanostructures on flame retardancy, smoke suppression, and dye adsorption behavior of flexible polyurethane foams. Polym. Degrad. Stab..

[179] Peng H.-K., Wang X.X., Li T.-T., Huang S.-Y., Lin Q., Shiu B.-C., Lou C.-W., Lin J.-H. (2018). Effects of hydrotalcite on rigid polyurethane foam composites containing a fire retarding agent: Compressive stress, combustion resistance, sound absorption, and electromagnetic shielding effectiveness. RSC Adv..

[180] Zheng X., Wang G., Xu W. (2014). Roles of organically-modified montmorillonite and phosphorous flame retardant during the combustion of rigid polyurethane foam. Polym. Degrad. Stab..

[181] Chen Y., Li M., Hao F., Yang C. (2022). Enhanced flame retardant performance of rigid polyurethane foam by using the modified OMMT layers with large surface area and ammonium polyphosphate. Mater. Today Commun..

[182] Mohammadi A., Wang D.-Y., Hosseini A.S., De La Vega J. (2019). Effect of intercalation of layered double hydroxides with sulfonate-containing calix[4]arenes on the flame retardancy of castor oil-based flexible polyurethane foams. Polym. Test..

[183] Zhang X., Wen Y., Li S., Wang Z., Xie H. (2021). Fabrication and characterization of flame-retardant and smoke-suppressant of flexible polyurethane foam with modified hydrotalcite. Polym. Adv. Technol..

[184] Kurańska M., Barczewski M., Uram K., Lewandowski K., Prociak A., Michałowski S. (2019). Basalt waste management in the production of highly effective porous polyurethane composites for thermal insulating applications. Polym. Test..

[185] Kairytė A., Kremensas A., Vaitkus S., Członka S., Strąkowska A. (2020). Fire Suppression and Thermal Behavior of Biobased Rigid Polyurethane Foam Filled with Biomass Incineration Waste Ash. Polymers.

[186] Kuźnia M., Magiera A., Zygmunt-Kowalska B., Kaczorek-Chrobak K., Pielichowska K., Szatkowski P., Benko A., Ziąbka M., Jerzak W. (2021). Fly Ash as an Eco-Friendly Filler for Rigid Polyurethane Foams Modification. Materials.

[187] Brannum D.J., Price E.J., Villamil D., Kozawa S., Brannum M., Berry C., Semco R., Wnek G.E. (2019). Flame-Retardant Polyurethane Foams: One-Pot, Bioinspired Silica Nanoparticle Coating. ACS Appl. Polym. Mater..

[188] Dong Q., Chen K., Jin X., Sun S., Tian Y., Wang F., Liu P., Yang M. (2019). Investigation of Flame Retardant Flexible Polyurethane Foams Containing DOPO Immobilized Titanium Dioxide Nanoparticles. Polymers.

[189] Li M.-E., Wang S.-X., Han L.-X., Yuan W.-J., Cheng J.-B., Zhang A.-N., Zhao H.-B., Wang Y.-Z. (2019). Hierarchically porous SiO2/polyurethane foam composites towards excellent thermal insulating, flame-retardant and smoke-suppressant performances. J. Hazard. Mater..

[190] Zheng X., Dong Q., Wang X., Yu P., Wang W., Zhang J., Ren L. (2021). Improvement of Flame Retardancy of Polyurethane Foam Using DOPO-Immobilized Silica Aerogel. Front. Mater..

[191] Díaz-Gomez A., Godoy M., Berrio M.E., Ramirez J., Jaramillo A.F., Medina C., Montaño M., Meléndrez M.F. (2022). Evaluation of the Mechanical and Fire Resistance Properties of Rigid Tannin Polyurethane Foams with Copper Oxide Nanoparticles. Fibers Polym..

[192] Vo D.K., Do T.D., Nguyen B.T., Tran C.K., Nguyen T.A., Nguyen D.M., Pham L.H., Nguyen T.D., Nguyen T.-D., Hoang D. (2022). Effect of metal oxide nanoparticles and aluminum hydroxide on the physicochemical properties and flame-retardant behavior of rigid polyurethane foam. Constr. Build. Mater..

[193] Xu Z., Chu F., Luo X., Jiang X., Cheng L., Song L., Hou Y., Hu W. (2022). Magnetic Fe_3_O_4_ Nanoparticle/ZIF-8 Composites for Contaminant Removal from Water and Enhanced Flame Retardancy of Flexible Polyurethane Foams. ACS Appl. Nano Mater..

[194] Xie H., Yang W., Yuen A.C.Y., Xie C., Xie J., Lu H., Yeoh G.H. (2017). Study on flame retarded flexible polyurethane foam/alumina aerogel composites with improved fire safety. Chem. Eng. J..

[195] Lin B., Yuen A.C.Y., Li A., Zhang Y., Chen T.B.Y., Yu B., Lee E.W.M., Peng S., Yang W., Lu H.-D. (2020). MXene/chitosan nanocoating for flexible polyurethane foam towards remarkable fire hazards reductions. J. Hazard. Mater..

